# Potential distribution of dominant malaria vector species in tropical region under climate change scenarios

**DOI:** 10.1371/journal.pone.0218523

**Published:** 2019-06-19

**Authors:** Godwin E. Akpan, Kayode A. Adepoju, Olakunle R. Oladosu

**Affiliations:** 1 African Regional Centre for Space Science and Technology Education in English (ARCSSTEE), Obafemi Awolowo University (OAU), Ile-Ife, Osun State, Nigeria; 2 Department of Geography, University of The Free State, Qwaqwa Campus, Qwaqwa, Phuthaditjhaba, South Africa; University of Molise, Isernia, ITALY

## Abstract

Risk assessment regarding the distribution of malaria vectors and environmental variables underpinning their distribution under changing climates is crucial towards malaria control and eradication. On this basis, we used Maximum Entropy (MaxEnt) Model to estimate the potential future distribution of major transmitters of malaria in Nigeria—*Anopheles gambiae* sensu lato and its siblings: *Anopheles gambiae* sensu stricto, and *Anopheles arabiensis* under low and high emissions scenarios. In the model, we used mosquito occurrence data sampled from 1900 to 2010 alongside land use and terrain variables, and bioclimatic variables for baseline climate 1960–1990 and future climates of 2050s (2041–2060) and 2070s (2061–2080) that follow RCP2.6 and RCP8.5 scenarios. The *Anopheles gambiae* species are projected to experience large shift in potential range and population with increased distribution density, higher under high emissions scenario (RCP8.5) and 2070s than low emission scenario (RCP2.6) and 2050s. *Anopheles gambiae* sensu stricto and *Anopheles arabiensis* are projected to have highest invasion with 47–70% and 10–14% percentage increase, respectively in Sahel and Sudan savannas within northern states in 2041–2080 under RCP8.5. Highest prevalence is predicted for Humid forest and Derived savanna in southern and North Central states in 2041–2080; 91–96% and 97–99% for *Anopheles gambiae* sensu stricto, and 67–71% and 72–75% for *Anopheles arabiensis* under RCP2.6 and RCP8.5, respectively. The higher magnitude of change in species prevalence predicted for the later part of the 21st century under high emission scenario, driven mainly by increasing and fluctuating temperature, alongside longer seasonal tropical rainfall accompanied by drier phases and inherent influence of rapid land use change, may lead to more significant increase in malaria burden when compared with other periods and scenarios during the century; especially in Humid forest, Derived savanna, Sahel and Sudan savannas.

## Introduction

With nearly half of the world's population at risk in 2017 [1], malaria continues as one of the highest killer infectious diseases in the world after lower respiratory tract infections, diarrhoeal diseases, human immunodeficiency virus / acquired immunodeficiency syndrome (HIV/AIDS) and tuberculosis, particularly among countries in the tropical region of the world [2]. According to World malaria report 2018 [3], an estimated 435,000 people died from malaria in 2017 from 219 million estimated global cases, with highest vulnerability among under-fives. Almost half of all global cases were accounted for by five tropical countries: Nigeria (25%), Democratic Republic of the Congo (11%), Mozambique (5%), India (4%) and Uganda (4%) [1,3]. With much efforts and considerable investments towards malaria control and eradication notwithstanding, some of the most plagued countries including Nigeria, India, Niger, Ethiopia, Madagascar, Myanmar, and Sudan (all within the tropical region), are reportedly below the operational universal coverage target of one insecticide treated nets (ITN) per two persons at malaria risk, together with lack of access to effective malaria treatment by large percentage of their population at risk, predominantly those living in rural remote settlements [3]. Malaria burden driven by poor vector control and inadequacy / inaccessibility of preventive material is expected to increase under changing world climates. Especially climate change resulting from the enhanced greenhouse effect [4] together with the direct effect of increased urbanisation / land use [5] and population drivers, is expected to produce changes in species distribution including that of malaria vectors in tropical ecosystems [6–9]. Shifts and increased prevalence induced by anthropogenic drivers [10,11] on vectors distribution may exacerbate human exposure to malaria infection [12].

**Fig 1:**
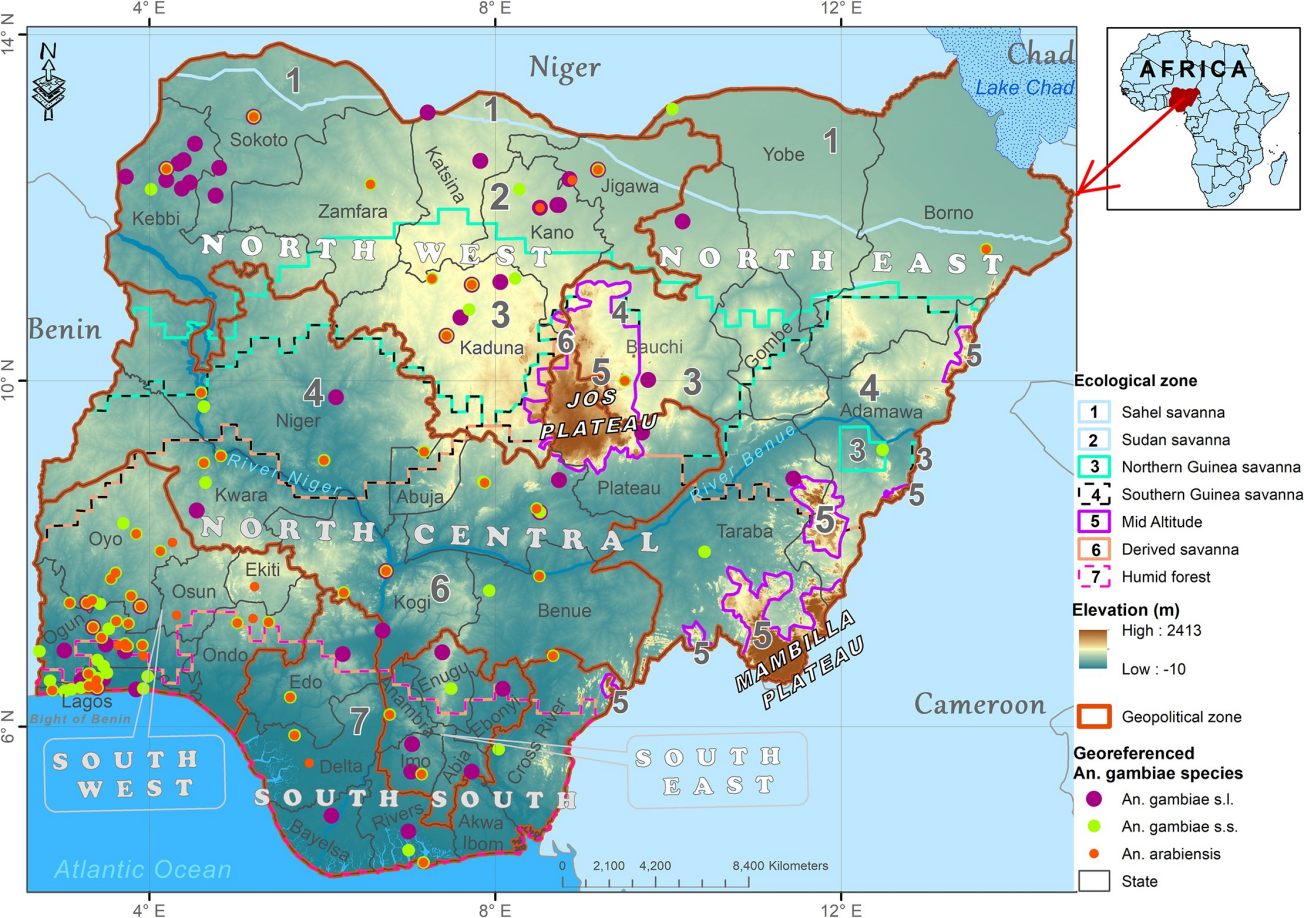
Map of Nigeria with georeferenced sampling points of *Anopheles gambiae* species, showing topographic relief, ecological, regional and state boundaries. *Anopheles* species sampling points reprinted for illustrative purposes only from Okorie *et al*. [48] under a CC BY 4.0 license, with permission from PLOS ONE [38].

The anthropogenic greenhouse gases (GHG) emission which indirectly impact species distribution is expected to continue throughout the 21st century in the absence of climate change policy, giving rise to what is described as ‘high emission scenario’ under the Representative Concentration Pathway 8.5 (RCP8.5) [13,14]. Basically, there are four RCP scenarios describing GHGs emissions and concentrations up to 2100 based on radiative forcing levels associated with assumptions around different combinations of land use, economic, technological (energy), demographic, policy, and institutional futures [15]. Among two stabilisation scenarios (RCP4.5 and RCP6), are one mitigation (low emissions) scenario (RCP2.6) and the high emissions scenario (RCP8.5) [15]. RCP 2.6 assumes global annual GHG emissions peak by 2020 and decline substantially, as global population peaks mid-century, and the use of non-fossil fuel increases with high global economic growth, resulting in low GHG emissions in the presence of climate policy [15–18]. Both low and high emissions scenarios are expected to lead to noticeable variations in regional (and sub-regional) temperatures and precipitation, which may impel changes in geographic distributions of malaria vectors and malaria risk pattern [19–22]. Within the tropical region, temperature is projected to rise by 3–6°C under RCP8.5 in West Africa by the end of the 21st century, just as total precipitation will change slightly with longer rainy season accompanied by drier phases [23]. This is predicted to be more diverse within regions and subregions of West African countries, especially in Nigeria due to ecosystem diversity in its regional and ecological zones [20,23]. A general decline in precipitation is predicted for Sudan, Sahel and Guinea savanna areas of North West, North East and North Central subregions, and a relatively large increase in precipitation in the coastal south [19], Humid Forest (within South South, South East and South West subregions), Derived savanna (within North Central, South east, South West and North East subregions), and highlands within Mid Altitude zone (see Fig 1 for the ecological and regional zones) [20,23–25]. Overall, temperature is projected to rise across the country by about 4.9°C with hotter nights and high humidity under RCP8.5 and limited to about 1.4°C under RCP2.6 on average from 1990 to 2100 [8,19,23]. The temperature in Humid Forest and Derived savanna under RCP8.5 is projected to increase by 3°C–4°C, and between 3°C and above 5°C in Guinea, Sudan and Sahel savannas in the north [20].

Altered rainfall pattern and elevated temperatures are expected to exacerbate climate extreme events and disasters such as flood, drought, sea level rise, landslides, strong winds, and proliferation of infectious disease vectors / disease transmission [9,19,26,27]. Over the course of the 21st century, several models have been used in predicting the impact of climate change across a variety of sectors and regions for risk analysis and adaptation strategies [28–31]. With respect to malaria risk assessment, several studies have been carried out on the impact of changing climate scenarios on the occurrence and distribution of malaria vectors across the world. Most of these studies focused on *Anopheles gambiae* sensu stricto (s.s.) and *Anopheles arabiensis* which are major siblings of the species complex—*Anopheles gambiae* sensu lato (s.l.) [12,32,33–37]. *An*. *gambiae* s.s. and *An*. *arabiensis* are dominant most efficient vector species of human malaria in the Afrotropical Region [38–41]. Tonnang *et al*. [35] used CLIMEX simulation model to predict possible shifts in *An*. *gambiae* s.s. and *An*. *arabiensis* boundaries southward and eastward of Africa rather than jumps into quite different climatic environments under climate change scenarios. This was previously observed by Peterson [34] who applied ecological niche model, Genetic Algorithm for Rule-set Prediction (GARP) to predict potential distributional shift from west to east and west to south of Africa for *An*. *gambiae* s.s. and *An*. *arabiensis*, respectively [12]. Drake and Beier [37] used Low Bias Bagging for One-Class-Classification (LOBAG-OC) to project extensive reductions in *An*. *arabiensis* habitat (as a result of climate change) in Western and Central Africa, and parts of some Eastern, North-eastern and Southern African countries. Also, under three climate scenarios, Ren *et al*. [32] used maximum entropy algorithm (MaxEnt) to predict prospective changes (increases) in future distribution of the four dominant vectors in China, different in each climate change scenario.

Previous studies demonstrated the distribution of *Anopheles* species under changing climate scenarios more in terms of general spatial distribution across bioclimatic domains within areas of interest, with little or no consideration of the influence of ecological subregions [12,32,33–37]. In this study we estimate future spatial distribution of dominant malaria vectors and relative climate impacts under low and high emissions scenarios that may affect malaria risk pattern under different ecosystem in tropical region, using a case study of Nigeria. This study builds on an earlier study [38] by assessing prospective changes in the distribution of *An*. *gambiae* sensu lato and its two major siblings: *An*. *gambiae* sensu stricto, and *An*. *arabiensis* across bioclimatic domains within ecological zones and administrative regions in the country, in relation to elemental conditions under both low and high emissions scenarios in 2041–2060 and 2061–2080, respectively. The choice of low and high emissions scenarios may have implications for policies towards the type and level of preparedness and intervention needed to reduce malaria burden caused by malaria vectors within states and regions along ecological gradients, in a world with and without climate policies [15,28,29]. MaxEnt, a well-established and successful method for species distribution modelling [42] was used based on its reliable usage and high performance among other modelling methods [43] for estimating disease vectors distribution and disease transmissions [32,44–47].

## Materials and methods

### Study area and mosquito occurrence data

Georeferenced *An*. *gambiae* species data (Fig 1) sampled between 1900 and 2010 across Nigeria (study area) was obtained from Nigeria *Anopheles* vector database (https://doi.org/10.1371/journal.pone.0028347.s001) [48]. Nigeria is located in the tropical region within Latitudes 4^o^ and 14^o^ north of the Equator and between Longitudes 2^o^ 2' and 14^o^ 30' east of the Greenwich Meridian (Fig 1) [20,38]. It has 36 regional states with Federal Capital Territory, Abuja within six broad geopolitical regions (Fig 1). Species distribution is distinctively defined by ecological zones on the basis of combinations of soil, landform and climatic characteristics in the country [38,49,50]. Climates are tropical at the coastal south within Humid forest and Derived savanna, sub-tropical further inland within Derived and Guinea savannas, semi-arid in the far north within Sudan and Sahel savannas, and temperate within Mid Altitude zone of Jos and Mambilla plateaus (Fig 1) [38,51]. Mean annual temperature ranges from 26°C to 33°C [20,23,50], contingent on climate zone. The north records rainfall between 500 mm and 750 mm annually, while the south records between 1,200 mm and above 4000 mm [23,38,51]. Mean elevation within the south is about 150 m above sea level, 600–700 m in the north, and 1,500–2,100 m within Mid Altitude zone (Fig 1) [20,38,52]. The country has over 190 million people [53] with about 90% at risk of malaria [54], estimated to be approximately 411 million and 794 million people in 2050 and 2100 [53,55] with 95% and above 97% of the populations projected to be at the risk of malaria, respectively [8]. Its urban population is expected to increase from 51.9% in 2019 to 72% in 2050 [55], with deforestation rate of about 3.5% per year [56].

### Environmental variables

To model impact of climate change under low and high emissions scenarios on malaria vector species distribution in Nigeria, nineteen bioclimatic variables with about 1 km^2^ spatial resolution were obtained for 1960–2080 from WorldClim (http://www.worldclim.org)—Global Climate Data, the global climate models (GCM)—community climate system model version 4 (CCSM4) based on RCP2.6 and RCP8.5 [57]. The bioclimatic variables were: annual mean temperature (bio1), mean diurnal range (bio2), isothermality (bio3), temperature seasonality (bio4), maximum temperature of warmest month (bio5), minimum temperature of coldest month (bio6), temperature annual range (bio7), mean temperature of wettest quarter (bio8), mean temperature of driest quarter (bio9), mean temperature of warmest quarter (bio10), mean temperature of coldest quarter (bio11), annual precipitation (bio12), precipitation of wettest month (bio13), precipitation of driest month (bio14), precipitation seasonality (coefficient of variation) (bio15), precipitation of wettest quarter (bio16), precipitation of driest quarter (bio17), precipitation of warmest quarter (bio18), and precipitation of coldest quarter (bio19). The climate data was based on three climate periods: the baseline time period 1960–1990 which observed data interpolations is referred to as current conditions [57–59], and future climate periods 2041–2060 (2050s) and 2061–2080 (2070s) [57]. The 1960–1990 Climate Normals used in this study from WorldClim [57] serves as an implicit predictor of the conditions characteristic of the future up to 2005 when it was created. It also serves as a stable benchmark against which changes in climate observations in 2041–2060 and 2061–2080 are compared [59]. Also incorporated in the model were land use land cover (lulc) data obtained from U.S. Geological Survey data release [60], and Digital Elevation Model (DEM) obtained from the Consultative Group on International Agricultural Research—Consortium for Spatial Information (CGIAR-CSI) [38,61].

### Modelling procedures and data analysis

Using Maximum entropy algorithm (MaxEnt) model version 3.3.3k [62], model operation was performed following the procedures in our previous study [38]. As a general-purpose machine learning technique of ecological niche modelling, MaxEnt estimates potential species distribution using species presence-only records and environmental layers [42,63]. In order to predict future occurrence and distribution of the *Anopheles* species, projected bioclimatic / environmental variables representing 2041–2060 (2050s) and 2061–2080 (2070) were added to projection directory in MaxEnt. Using all available data without having an independent dataset under sub-sample replicated run type, occurrence data for each species was split twenty-one times into training (75%) and testing (25%) subsets; performed to test the predictiveness / performance of the MaxEnt model specified by Area Under the Receiver Operating Characteristic (ROC) Curve (AUC) [38]. The AUC measures the ability of the model to discriminate between sites where a species is present (***y = 1***) against where it is absent (***y = 0***), given as a plot of sensitivity against specificity [38,64–66]. The AUC was considered more in terms of model’s predictiveness, which according to Lobo *et al*. [67], the value of AUC tells of the degree to which a species is restricted along the range of predictor conditions in the study area, in order that presences can be told apart from absences [43]. To guard against bias in datasets, following large ranges of the documented species [64] relative to the study area (especially in the North Eastern part of the country) (Fig 1) [38], a bias layer was created to provide MaxEnt with background samples (pseudo-absences) [43,68–70]; defining locations with documented *Anopheles* species as 1, and “no data” in the grid of unsampled pixels [71]. More environmentally distant pseudo-absences from the presences (as applied in this study) increases the rate of well-predicted absences and the AUC scores [67]. Over-prediction / under-prediction of the relationships by the model was controlled by increasing maximum iterations from 500 (default) to 5000, allowing the model to have adequate time for convergence; and regularization was left at 1 (default) to minimise model over-fitting [38,43,65].

The combined environmental variables predicted the probability of species occurrence by producing a point-wise mean (model images), created by MaxEnt model based on maximum entropy of optimal conditions of included environmental variables that match the threshold value within species occurrence records [38,65]. These images were classified in ArcMap for distributions of the studied mosquito species, and reclassified into suitable and unsuitable habitats using 10 percentile training presence logistic threshold provided by MaxEnt. Suitable habitat was defined to include 90% of the data used to develop the model, to take care of some errors inherent in the datasets [38,65]. The mean distribution density of each *Anopheles* species was determined from zonal statistics in ArcGIS, across ecological and geopolitical zones, and in each state [38]. Prevalence (distribution density in percent) was derived from the mean distribution density according to the equation, **p**_**x**_ **= (y**_**x**_**/y**_**max**_**)100;** where **p**_**x**_ represents species prevalence in year **x**, **y**_**x**_ represents predicted mean distribution density of species in year **x**, and **y**_**max**_ represents maximum mean distribution density of species estimated by zonal statistics. Mean distribution density less than or equal to one (equivalent to ≤50% prevalence) defines the probability of the species not occurring, greater than one (equivalent to >50% prevalence) defines species presence, and equal to two (equivalent to 100% prevalence) represents maximum prevalence of species within a zone or state [38]. Percentage change (**y**) of species between baseline and future climates was derived from the equation **y = ((y**_**x**_**-y**_**0**_**)/y**_**0**_**)100**, where **y**_**0**_ represents species prevalence under baseline climate, **y**_**x**_ represents species prevalence in year **x**. The contribution of environmental variables to the delineation of suitable environments for the *Anopheles* species were examined using jackknife test of variable importance and analysis of percent contribution of each variable [38,45]. In Jackknife test the training gained by each variable is estimated as if the model was run with one variable, and then compare it to the training gain involving all variables [38,65]. Logical assessment of variables contributions was achieved by evaluating estimates of relative contributions of the environmental variables alongside jackknife plots produced for training gain, test gain and AUC (S1 File) [38,64].

## Results

### Potential future distributions of *An*. *gambiae* species under RCP2.6 and RCP8.5

**Fig 2:**
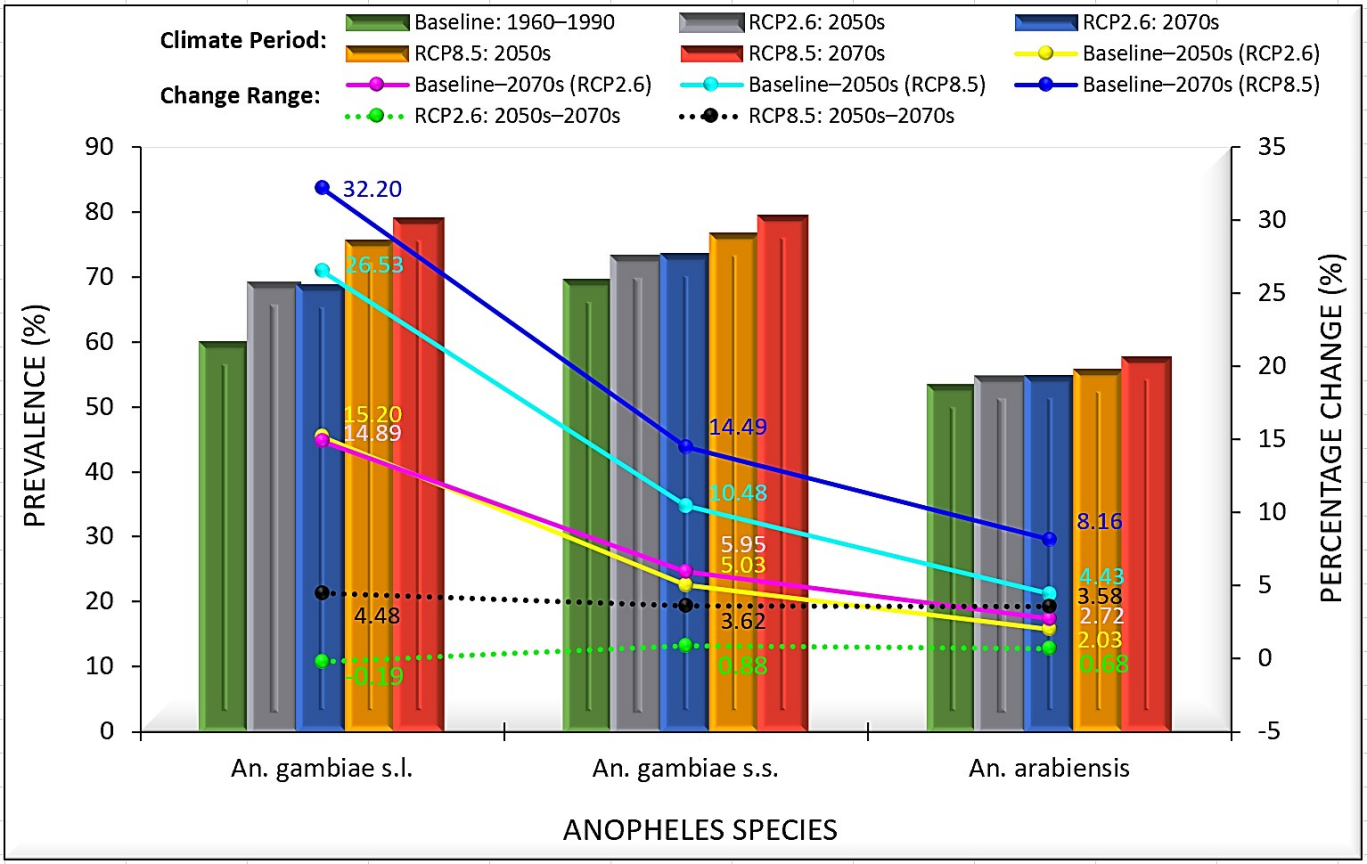
Prevalence of *An*. *gambiae* species under low and high emissions scenarios and percentage change across all bioclimatic domains in Nigeria. RCP2.6 represents low emissions scenario and RCP8.5 represents high emissions scenario.

**Fig 3:**
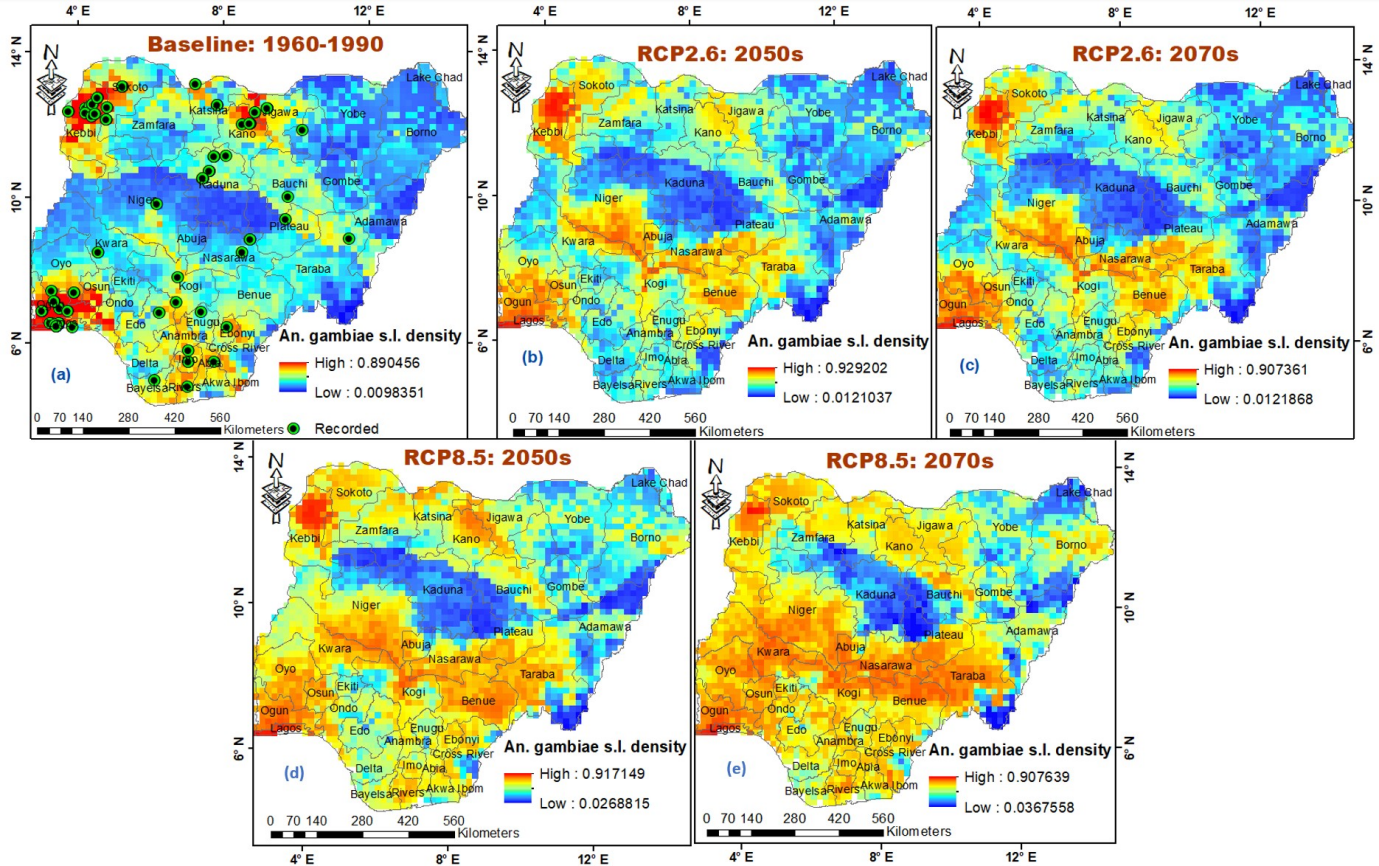
Potential occurrence and distribution of *An*. *gambiae* s.l. in Nigeria. (a) baseline climate 1960–1990, (b) low emissions scenario (RCP2.6) 2041–2060, (c) low emissions scenario (RCP2.6) 2061–2080, (d) high emissions scenario (RCP8.5) 2041–2060 and (e) high emissions scenario (RCP8.5) 2061–2080. *Anopheles* species sampling points reprinted for illustrative purposes only from Okorie *et al*. [48] under a CC BY 4.0 license, with permission from PLOS ONE [38].

**Fig 4:**
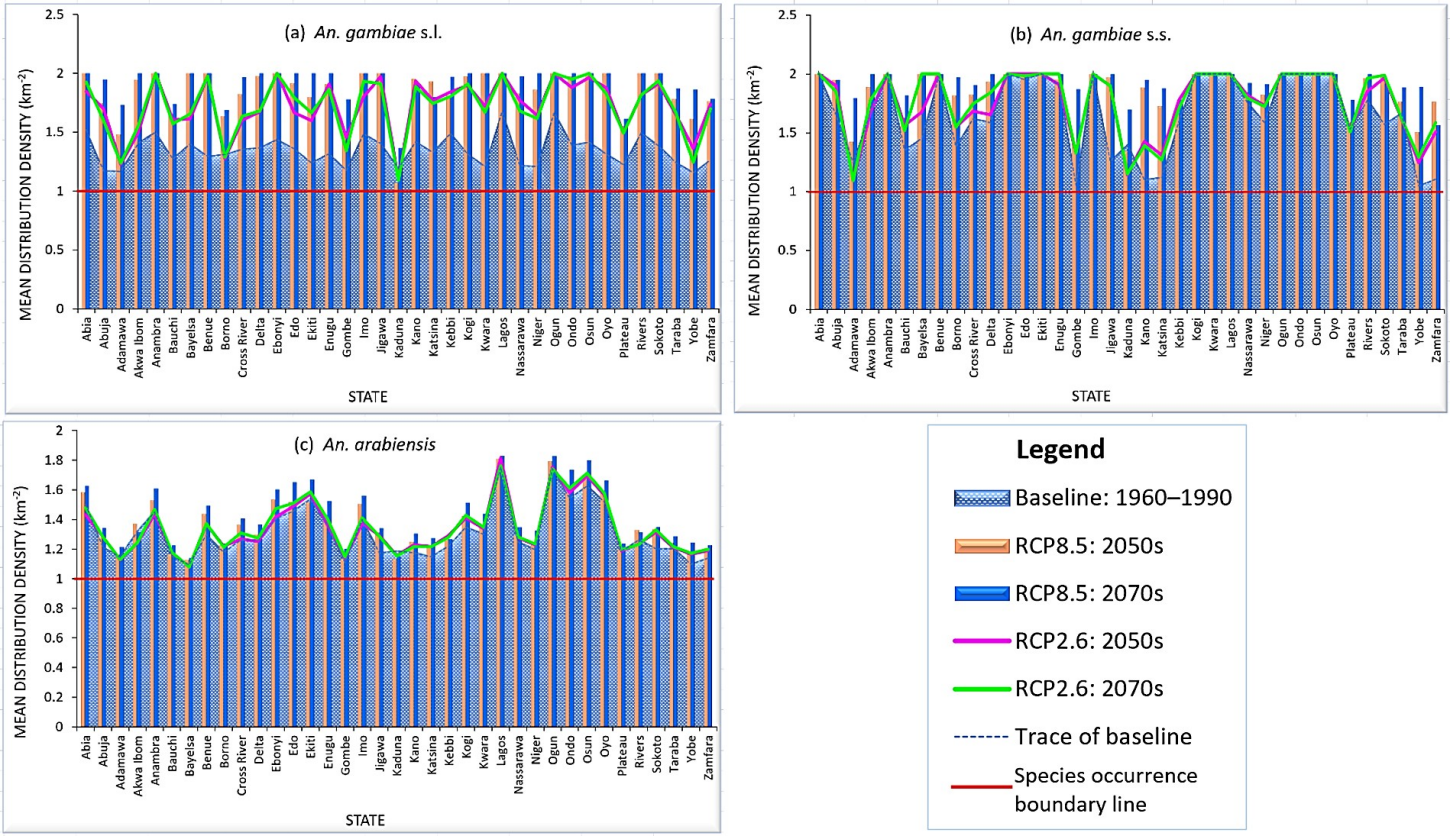
Mean distribution density of *An*. *gambiae* species in each Nigerian state under baseline climates 1960–1990 and low emissions scenario (RCP2.6) / high emissions scenario (RCP8.5) in 2041–2060 (2050s) and 2061–2080 (2070s). Note: red boundary line on the graph defines presence and absence conditions for each *Anopheles* species from zonal statistics; if 1 ≡ 0 (species does not occur), >1 = species occur, and 2 = maximum species prevalence [38].

Noticeable changes have been projected to occur in potential distribution of the dominant malaria vector species complex, *An*. *gambiae* s.l. and its two major siblings under the chosen climate change scenarios within bioclimatic and ecological domains in the tropical country—Nigeria. The ‘business as usual’ high emissions scenario (RCP8.5) without large investments threatens to increase the future geographic range, distribution density and prevalence of *An*. *gambiae* species more than the mitigation (low emissions) scenario (RCP2.6). *An*. *gambiae* s.l., a species complex of eight reproductively isolated species [40,41] is projected to increase across all bioclimatic domains in the country with total percentage increase of 26.53% and 32.20% under RCP8.5, and 15.20% and 14.89% under RCP2.6 in 2050s and 2070s, respectively (Fig 2). This is expected to translate into large range expansions and high prevalence with increased distribution density within Humid forest (36.51% and 39.70% under RCP8.5) and (19.19% and 23.30% under RCP2.6), Derived savanna (48.60% and 52.26% under RCP8.5) and (43.69% and 43.03% under RCP2.6), Sudan savanna (37.66% and 44.31% under RCP8.5) and (28.02% and 26.66% under RCP2.6), and Sahel savanna (47.725 and 54.13% under RCP8.5) and (24.99% and 22.96% under RCP2.6) in 2050s and 2070s, respectively (Table 1; Fig 3). The Southern Guinea savanna from Oyo state through Kwara state to Niger state (Fig 1) which appears less suitable for *An*. *gambiae* s.l. under current climates [38], is projected to experience large invasion with increased distribution density especially under RCP8.5 in 2050s (by 43.14%) and more increasingly so in 2070s (by 51.97%); increasing by 28.44% under RCP2.6 in 2050s but declines to 25.09% in 2070s (Table 1; Fig 3; S1 Fig). This scenario is projected to trigger a north-eastward species shift, making Kaduna state within Northern Guinea savanna least suitable for *An*. *gambiae* s.l. in 2050s and 2070s, against Yobe state within Sudano-Sahelian zone under current climates (Fig 4; S1 Fig). The north-eastward species shift is expected to cause *An*. *gambiae* s.l. invasion in the less suitable Sahel savanna especially in Sokoto and Katsina states, and parts of Yobe and Borno states. The less suitable north-eastern region landmass (currently) is projected to become more suitable for *An*. *gambiae* s.l. in 2050s and 2070s, especially under RCP8.5 (Fig 3; Table 2; S1 Fig). However, the Mid Altitude zone is projected to become less suitable for the *An*. *gambiae* complex under RCP2.6 with percentage decrease of 1.58% in 2050s and 1.44% in 2070s, and 0.66% in 2050s under RCP8.5, particularly the Mid Altitude areas of Jos plateau, Mambilla plateau and highlands in Adamawa, Borno and Cross River states (Table 1; Fig 3; S1 Fig). The *An*. *gambiae* s.l. may likely increase in population across the Mid Altitude zone by 6% in 2070s under RCP8.5 (Table 1). Also, highlands within Ondo, Ekiti, Edo, Kogi, Enugu and Anambra states are projected to be less suitable for *An*. *gambiae* s.l. between 2041 and 2060; but are projected to experience *An*. *gambiae* s.l. invasion between 2061 and 2080 under RCP8.5 (Fig 3; S1 Fig). By administrative regions (geopolitical zones), the South East and South West are projected to hit species maximum prevalence in 2070s under RCP8.5 (Table 2). Other regions are estimated to experience large increased mean distribution density in 2050s and larger in 2070s under RCP8.5. Prevalence is projected to be larger in 2050s in the three northern regions than in 2070s under RCP2.6 (Table 2). According to the model projections, fourteen states will hit maximum prevalence of *An*. *gambiae* s.l. in both 2050s and 2070s under high emission scenario (Fig 4). These states are: Abia, Akwa Ibom, Bayelsa, Benue, Ebonyi, Imo, Jigawa, Kwara, Lagos, Ogun, Osun, Oyo, Rivers and Sokoto (Fig 4). Other states are projected to experience a sequential increase in mean distribution density of *An*. *gambiae* s.l., with observed anomaly in Kaduna, Katsina and Kano states, where either current climates record higher density than 2050s/2070s, or 2050s record higher density than 2070s (Fig 4).

**Table 1:** Prevalence of *An*. *gambiae* species within ecological zones and percentage change under RCP2.6 and RCP8.5.

Ecological zone	Climate period / Prevalence (%)					Climate period / Percentage change (%)			
	Baseline: 1960–1990	RCP2.6		RCP8.5		RCP2.6		RCP8.5	
		2050s	2070s	2050s	2070s	2050s	2070s	2050s	2070s
	(a) *An*. *gambiae* s.l.								
Sahel savanna	59.38	74.22	73.01	87.72	91.52	24.99	22.96	47.72	54.13
Sudan savanna	67.21	86.04	85.13	92.52	96.99	28.02	26.66	37.66	44.31
Northern Guinea savanna	61.96	70.06	67.40	74.78	83.33	13.07	8.79	20.69	34.50
Southern Guinea savanna	60.06	77.14	75.13	85.97	91.28	28.44	25.09	43.14	51.97
Mid Altitude	58.45	57.53	57.61	58.06	61.96	-1.58	-1.44	-0.66	6.00
Derived savanna	65.18	93.66	93.23	96.86	99.25	43.69	43.03	48.60	52.26
Humid forest	71.47	85.18	88.12	97.56	99.83	19.19	23.30	36.51	39.70
	(b) *An*. *gambiae* s.s.								
Sahel savanna	57.71	75.78	76.82	90.14	98.62	31.30	33.10	56.18	70.87
Sudan savanna	58.96	76.55	76.46	86.95	93.89	29.83	29.68	47.48	59.24
Northern Guinea savanna	60.22	72.27	71.53	76.55	87.17	20.02	18.79	27.12	44.75
Southern Guinea savanna	61.88	78.57	77.60	85.06	93.75	26.98	25.41	37.47	51.51
Mid Altitude	59.02	61.83	60.33	63.44	75.54	4.76	2.21	7.49	28.00
Derived savanna	69.26	96.59	96.92	98.23	99.11	39.45	39.93	41.82	43.09
Humid forest	67.59	91.37	95.87	97.56	99.50	35.18	41.85	44.34	47.22
	(c) *An*. *arabiensis*								
Sahel savanna	56.12	60.61	60.90	62.76	63.97	8.01	8.52	11.84	13.99
Sudan savanna	58.08	61.69	61.91	62.16	64.14	6.22	6.59	7.02	10.44
Northern Guinea savanna	58.57	58.19	58.16	59.64	59.59	-0.64	-0.69	1.84	1.75
Southern Guinea savanna	59.82	60.75	60.83	61.95	64.52	1.56	1.69	3.57	7.86
Mid Altitude	57.90	56.87	56.93	58.98	56.87	-1.78	-1.68	1.87	-1.78
Derived savanna	68.24	70.51	71.10	73.66	75.47	3.32	4.19	7.93	10.59
Humid forest	67.79	67.33	68.56	71.62	74.08	-0.68	1.13	5.65	9.27

**Note:** Species prevalence = 50% represents unsuitable zone and species absence, designated with a green square; >50% represents suitable zone and species presence; and 100% represents highly suitable zone with maximum prevalence of species, designated with a red square [38]. RCP2.6 represents low emissions scenario and RCP8.5 represents high emissions scenario.

**Table 2:** Prevalence of *An*. *gambiae* species within regional zones under RCP2.6 and RCP8.5.

*Anopheles* species	Climate period	Regions / prevalence (%)					
		South South	South East	South West	North Central	North East	North West
***An*. *gambiae* s.l.**	Baseline: 1960–1990	69.10	71.94	71.29	61.75	59.79	67.16
	RCP2.6: 2050s	82.53	95.73	94.49	86.24	71.52	85.39
	RCP2.6: 2070s	85.16	96.95	94.44	84.87	70.17	83.97
	RCP8.5: 2050s	96.37	98.82	99.10	93.59	81.87	86.52
	RCP8.5: 2070s	99.59	100.00	100.00	97.39	88.71	90.05
***An*. *gambiae* s.s.**	Baseline: 1960–1990	64.46	71.12	78.28	64.85	58.48	60.07
	RCP2.6: 2050s	88.76	98.78	100.00	91.44	70.29	78.20
	RCP2.6: 2070s	94.11	100.00	100.00	91.51	69.99	77.80
	RCP8.5: 2050s	96.57	99.41	100.00	93.74	80.40	86.36
	RCP8.5: 2070s	98.77	100.00	100.00	96.79	94.02	89.97
***An*. *arabiensis***	Baseline: 1960–1990	64.65	70.75	79.70	62.51	57.05	58.95
	RCP2.6: 2050s	64.63	70.02	81.59	64.48	58.69	61.82
	RCP2.6: 2070s	65.76	72.13	81.96	64.85	58.98	61.77
	RCP8.5: 2050s	69.63	77.21	86.00	65.50	59.00	60.00
	RCP8.5: 2070s	70.70	78.96	86.79	69.07	61.99	63.03

**Note:** Species prevalence = 50% represents species absence, designated with a green square; >50% represents species presence; and 100% represents maximum prevalence of species, designated with a red square [38]. RCP2.6 represents low emissions scenario and RCP8.5 represents high emissions scenario.

**Fig 5:**
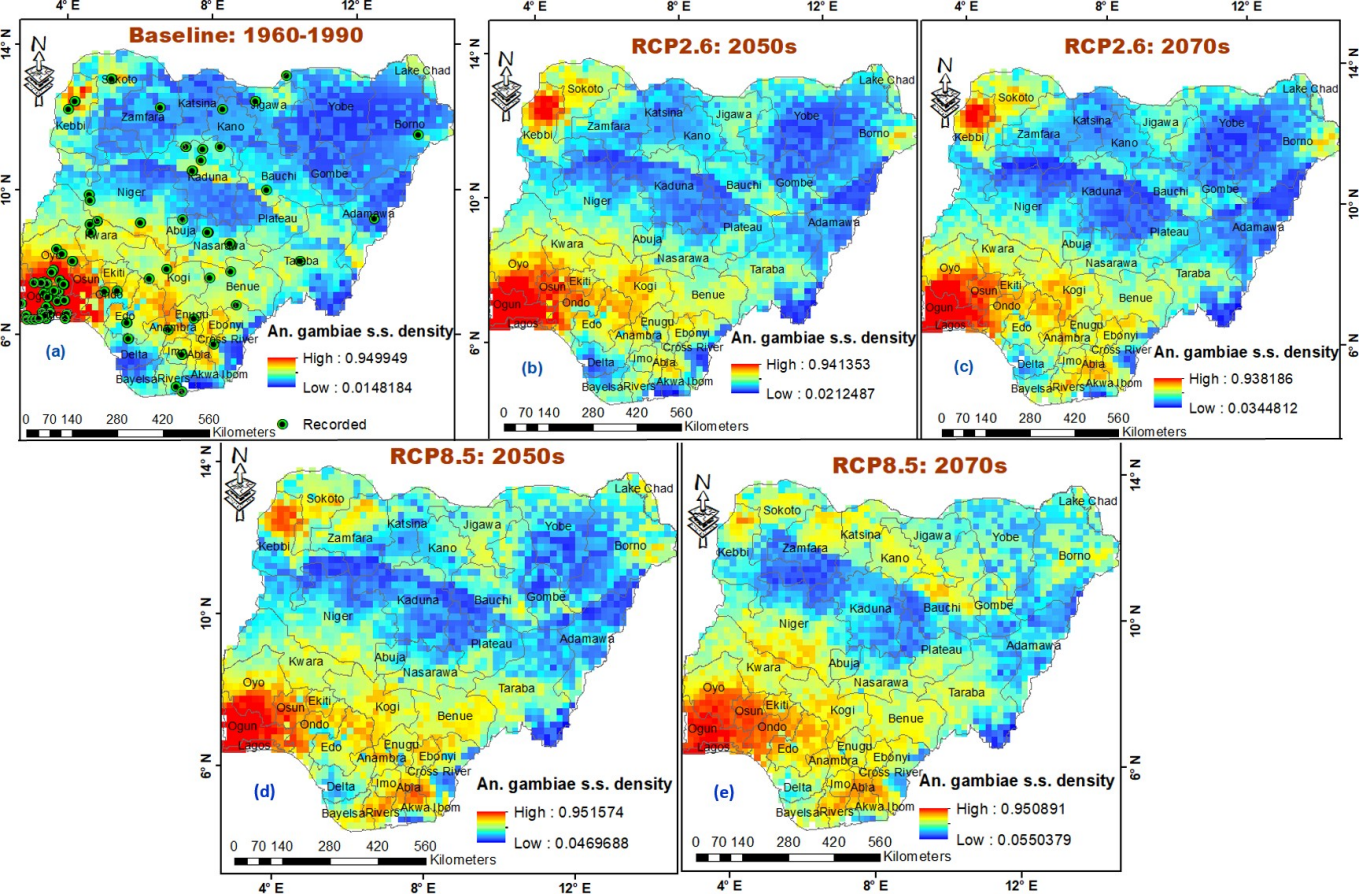
Potential occurrence and distribution of *An*. *gambiae* s.s. in Nigeria. (a) baseline climate 1960–1990, (b) low emissions scenario (RCP2.6) 2041–2060, (c) low emissions scenario (RCP2.6) 2061–2080, (d) high emissions scenario (RCP8.5) 2041–2060 and (e) high emissions scenario (RCP8.5) 2061–2080. *Anopheles* species sampling points reprinted for illustrative purposes only from Okorie *et al*. [48] under a CC BY 4.0 license, with permission from PLOS ONE [38].

The mean distribution density of *An*. *gambiae* s.s. is projected to increase from current climates across all bioclimatic domains in the country by 10.48% and 14.49% in 2050s and 2070s under high emissions scenario, and by 5.03% and 5.95% under low emission scenario, respectively (Fig 2). This is expected to lead to large geographic range expansion and increased prevalence (percentage increase) within Derived savanna (41.82% and 43.09% under RCP8.5) and (39.45% and 39.93% under RCP2.6), Humid forest (44.34% and 47.22% under RCP8.5) and (35.18% and 41.85% under RCP2.6), and mostly west of Sudan savanna (47.48% and 59.24% under RCP8.5) and (29.83% and 29.68% under RCP2.6) in 2050s and 2070s, respectively (Table 1; Fig 5; S2 Fig). *An*. *gambiae* s.s. is expected to maintain its maximum prevalence even beyond 2080 in all states within South Western and South Eastern regions (Tables 1 and 2). Like *An*. *gambiae* s.l. under high emissions scenario, *An*. *gambiae* s.s. is projected to experience shift in composition and geographic range in Niger state; as boundaries (areas) along Zamfara, Kaduna, Bauchi and Plateau states within Northern and Southern Guinea savannas, and Mid Altitude seem less suitable for *An*. *gambiae* s.s. in 2050s, and less so in 2070s (Fig 5; S2 Fig). North-eastward species shift is expected to lead to widespread of *An*. *gambiae* s.s. in the less suitable Sahel savanna by 56.18% and 70.87% under RCP8.5, and 31.30% and 33.10% under RCP2.6 in 2050s and 2070s, respectively, with less suitable east of Sudan savanna experiencing high prevalence of *An*. *gambiae* s.s. in 2070s under RCP8.5 (Table 1). This will lead to species invasion and increased distribution density in the less suitable North-Eastern landmass, especially within Lake Chad areas of Borno state (Table 1; Fig 5; S2 Fig). All other states are estimated to experience sequential increases in mean distribution density of *An*. *gambiae* s.s. in 2050s and 2070s, respectively. Observed anomaly is estimated to occur in Kaduna, Sokoto and Zamfara states under RCP8.5, just as it occurs in many other states under RCP2.6, either from higher distribution density under current climates than 2050s/2070s, or in 2050s than 2070s (Figs 4 and 5).

**Fig 6:**
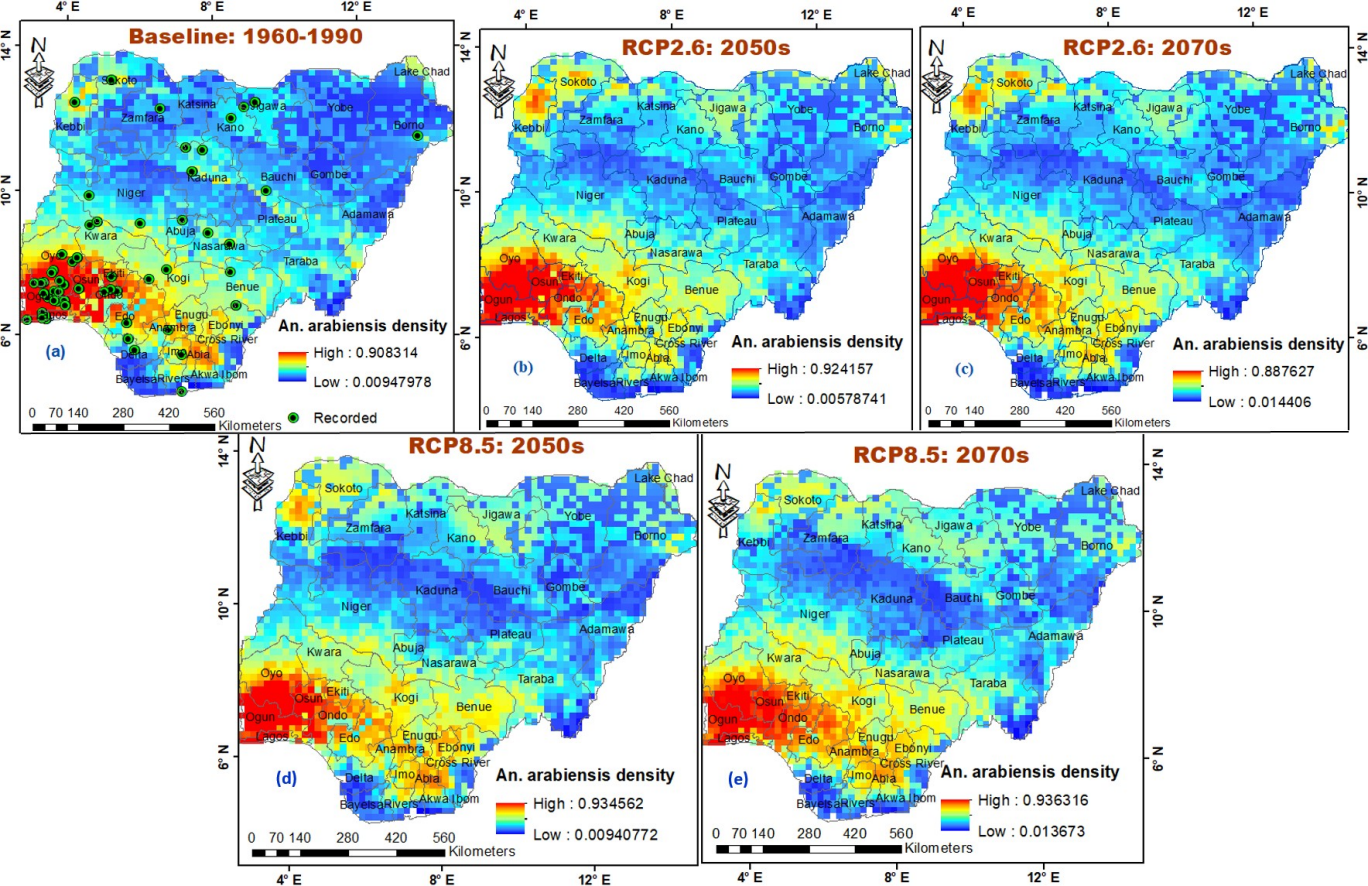
Potential occurrence and distribution of *An*. *arabiensis* in Nigeria. (a) baseline climate 1960–1990, (b) low emissions scenario (RCP2.6) 2041–2060, (c) low emissions scenario (RCP2.6) 2061–2080, (d) high emissions scenario (RCP8.5) 2041–2060 and (e) high emissions scenario (RCP8.5) 2061–2080. *Anopheles* species sampling points reprinted for illustrative purposes only from Okorie *et al*. [48] under a CC BY 4.0 license, with permission from PLOS ONE [38].

The mean distribution density of *An*. *arabiensis* is projected to increase across all bioclimatic domains in the country by 4.43% in 2050s and 8.16% in 2070s under high emissions scenario, and by 2.03% and 2.72% under low emissions scenario, respectively (Fig 2). This projected increase may lead to large geographic expansion range and increased prevalence of *An*. *arabiensis* within ecological zones; Derived savanna (7.93% and 10.59% under RCP8.5) and (3.32% and 4.19% under RCP2.6), Humid forest (5.65% and 9.27% under RCP8.5) and (-0.68% and 1.13% under RCP2.6), Sudan savanna (7.02% and 10.44% under RCP8.5) and (6.22% and 6.59% under RCP2.6), Sahel savanna (11.84% and 13.99% under RCP8.5) and (8.01% and 8.52% under RCP2.6), and Southern Guinea savanna (3.57% and 7.86% under RCP8.5) and (1.56% and 1.69% under RCP2.6) in 2050s and 2070s, respectively (Table 1; Fig 6). The probability of suitable areas for *An*. *arabiensis* in the less suitable Sahel and Sudan savannas is estimated to be high in 2050s, and higher in 2070s, especially under high emissions scenario (Table 1; Fig 6; S3 Fig). The model predicted that under high emissions scenario, *An*. *arabiensis* may likely go extinct in Kaduna South and parts of Kaduna North and Central within Northern Guinea savanna in 2050s, and shift towards north of Kaduna state, where environmental conditions may favour its occurrence around Kaduna boundary with Kano state in 2070s (Fig 6; S3 Fig). This species shift results in prevalence anomaly in Kaduna state under both RCP2.6 and RCP8.5 (Fig 4). Anomaly is also observed in Akwa Ibom, Ekiti, Kebbi and Rivers states under RCP8.5; and in Kano, Kebbi, Lagos and Ogun states under RCP2.6 (Fig 4). Mid Altitude zone of Jos and Mambilla plateaus, and highlands along Cameron boundary are projected to experience more presences of *An*. *arabiensis* in 2050s under RCP8.5 with percentage increase of 1.87%, but record less presences in 2070s with percentage decrease of 1.78% (Table 1; Fig 6; S3 Fig). Similarly, the *An*. *arabiensis* is projected to decrease in population within the Mid Altitude zone under RCP2.6 by 1.78% and 1.68% in 2050s and 2070s, respectively (Table 1; Fig 6; S3 Fig). The Fresh water and Mangrove swamp forests (within Humid forest) of the Niger Delta region are projected to remain less suitable for *An*. *arabiensis* under both RCP2.6 and RCP8.5 in 2050s and 2070s Especially in Bayelsa state where environmental variables least favour the occurrence of *An*. *arabiensis* (Fig 4). By regions, the model predicted that *An*. *arabiensis* will continue its highest prevalence in South West, followed by South East, South South, North Central, North West; and lowest in North East (Table 2).

Generally, large increase in population is projected for the studied *An*. *gambiae* species under RCP8.5 between 2050s and 2070s. With less than one percentage increase in prevalence, a decline or a likely state of dynamic equilibrium in vectors population is expected to be the case under RCP2.6 (Fig 2). Also, model projections show that sympatric species—*An*. *gambiae* s.s. and *An*. *arabiensis* [46,56] do not always occur together, nor with other members of the *An*. *gambiae* complex such as *An*. *coluzzii*, *An*. *melas*, etc. (Figs 2 and 4; S1–S3 Figs). *An*. *gambiae* s.s. is estimated as the most prevalent species of the *An*. *gambiae* complex. It is predicted to occur in many areas least suitable for other members of the complex, resulting in its higher prevalence than the *An*. *gambiae* s.l. In this study, *An*. *gambiae* s.l. is predicted as a complex species in areas suitable for one or more of its siblings other than only *An*. *gambiae* s.s. and/or *An*. *arabiensis* (Figs 2 and 4).

### Contributions of environmental variables to future distributions of *An*. *gambiae* species under RCP2.6 and RCP8.5

MaxEnt predicted that annual mean temperature (bio1), 33°C which may rise by about 4.9°C on average under RCP8.5 and 1.4°C under RCP2.6 [8,20,24], will greatly regulate (in isolation) the total energy inputs in the ecosystem towards high prevalence of *An*. *gambiae* s.l. between 2041 and 2080 in Nigeria (S1 File). Cold temperature anomalies throughout the year (bio6) is also projected to greatly influence year-round widespread of *An*. *gambiae* s.l. between 2041 and 2080, especially under RCP8.5 when combined with precipitation of driest quarter (bio17) (December–February, projected to decline by 18 mm, 15 mm and 10 mm in December, January and February, respectively, in the Humid forest and Derived savanna) [20] (S1 File). This is also estimated to be complemented by mean temperature of the wettest quarter (bio8) (June–August), temperature annual range (bio7) of more than 28°C–36°C by 3°C–5°C [20], mean temperature of the warmest quarter (bio10) (March–May), mean temperature of driest quarter (bio9), maximum temperature of warmest month (bio5) (about 35°C in February in the south and above 40°C in April in the north) [20,38], changes in land use land cover (lulc), and terrain surface (DEM) (S1 File). However, the occurrence and seasonal distribution pattern of *An*. *gambiae* s.l. is expected to be altered if precipitation of coldest quarter (bio19) (June–August, about 211 mm in the arid north and above 2000 mm in the coastal south) is omitted, because bio19 contains the most information absent in other variables.

Mean diurnal range (bio2) between 7°C and above 16°C [20,72] is projected to greatly regulate (in isolation) the distribution and prevalence of *An*. *gambiae* s.s. between 2041 and 2080 under both low and high emissions scenarios (S1 File). This is estimated to be complemented by precipitation (bio17) and mean temperature (bio9) of driest quarter, and precipitation of coldest (bio19) and warmest (bio18) quarters when used alongside all other environmental variables including terrain surface (DEM) (S1 File). Changes in land use and land cover dynamics (lulc) is estimated as the variable with the most information that is not present in the other variables, regarding the distribution and prevalence of *An*. *gambiae* s.s. as well as *An*. *arabiensis* in 2050s and 2070s (S1 File). Precipitation of driest quarter (bio17) is projected to highly influence (in isolation) the widespread and seasonal distributions of *An*. *arabiensis* between 2041 and 2080 under both low and high emissions scenarios (S1 File). Precipitation of coldest quarter (bio19), precipitation seasonality (bio15), mean diurnal range (bio2), mean temperature of driest quarter (bio9), precipitation of warmest quarter (bio18), precipitation of driest month (bio14), and temperature annual range (bio7) are expected to combine with all other environmental variables to greatly influence year-round occurrence and distribution of *An*. *arabiensis* in 2050s and 2070s, respectively (S1 File).

## Discussion

In relation to climate drivers, the potential future distribution of *An*. *gambiae* s.l. and its two major siblings: *An*. *gambiae* s.s. and *An*. *arabiensis* in Nigeria is highly worrisome. Climate change under both high and low emissions scenarios is expected to cause large geographic range expansions with increased distribution density, species shifts and invasions in areas previously too cool for the *Anopheles* species population [34,35]. Higher prevalence of the malaria vectors projected for high emission scenario corroborates with the prediction of ecological models about shift in the distribution of world biomes in response to changes in climate system associated with increased greenhouse gases [38,73]. Confirming earlier claim that areas with low presence of the studied *Anopheles gambiae* species will possibly experience high prevalence with species migration and invasion, in response to changes in environmental variables fundamental to their distribution patterns [38]. This is evidenced in the scenario that the less suitable areas of Sudan, Sahel, Northern and Southern Guinea savannas within Gombe, Bauchi, Yobe, Adamawa and Borno states in North Eastern region [38], are becoming more suitable for the occurrence and distribution of these *Anopheles* species under the changing climate regimes. This is expressed in longer rainy season and warmer nights that promote vectors proliferation in a continuous high emissions 21st Century’s world [20,25]. The general decline or near dynamic equilibrium in vector species population between 2050s and 2070s under the mitigation scenario—RCP2.6 is assumed to follow indirect effect of mitigation scenario, where increase in global mean temperature is limited to 2°C as regional mean temperature within the tropical country is limited to about 1.4°C [8,15,16]. Nevertheless, low environmental suitability has been projected to continue in some areas for *An*. *gambiae* s.l., *An*. *gambiae* s.s., and *An*. *arabiensis* based on topography and other ecological gradients [74,75]. This is of course highly noticeable in some high elevation areas of Kaduna, Zamfara, Kebbi, Niger, Bauchi, Gombe and Borno states within Northern and Southern Guinea savannas, and parts of Plateau, Adamawa, Bauchi, and Taraba states within Mid Altitude zone [38]. The projected scenario also supports the assertion by Siteti *et al*. [75], that, topographic and human settlement patterns affect the spatial distribution of malarial mosquitoes [38,76]. It is also apparent from this study that *An*. *gambiae* s.s. and *An*. *arabiensis* are likely to be more widespread with higher relative probability of occurrence in lowlands and areas with human settlements [77]. *An*. *arabiensis* exhibits low probability of occurrence in Fresh water and Mangrove swamp forests [12,33,35] along humid Atlantic coast of the Humid forest within South South region, either based on landforms [73,74], soil or climatic characteristics [38,49,50]; corroborating with Ayala *et al*. [77] who classified these zones unsuitable for *An*. *arabiensis* in Cameroon. However, *An*. *gambiae* s.s. is expected to continue its highest prevalence amongst other species, in line with Okwa *et al*. [78] that, *An*. *gambiae* s.s. is the most efficient and most widespread within the *gambiae* complex [38]. According to Olayemi *et al*. [79], the epidemiological success of *An*. *gambiae* s.s. is largely dependent on its highly dynamic ecological behaviour, which it evolved over a long time to take advantage of certain tropical weather conditions that encourage mosquito proliferation and human / vector contact.

The projected *An*. *gambiae* s.s. and *An*. *arabiensis* expand largely in range and shift in sympatric coexistence under changing future climates [12,46,56], in agreement with Peterson [34] whose future projections on suitable areas for these vectors in Africa match the suitability/occurrence patterns in bioclimatic domains within Nigeria. The distribution of these highly anthropophilic members of *Anopheles gambiae* complex appears relative to ecological zones within the tropical region [40]. The prediction of their dominant occurrence in humid and sub-humid (Derived and Guinea savannas) zones of Nigeria in West Africa (in this study) corresponds to the prediction of their environmental suitability in sub-humid zone in East Africa, from west of Mount Kilimanjaro to the coastal plain of northern Tanzania [47]. It similarly corresponds to their wide distribution in humid and sub-humid domains in Madagascar within the southeast coast of Africa [80]. Their distribution within these ecological zones in Nigeria also corresponds closely to expert-based predictions of Lindsay *et al*. [40], Levine *et al*. [46] and Moffett *et al*. [44], who presented climate suitability zones for these species. While the projected distribution of *An*. *gambiae* s.s. agrees with Tonnang *et al*. [12] regarding the region (Nigeria) in a similar study on Africa as Peterson [34], especially under their chosen climate scenario 1 (similar to RCP8.5), the proposed distribution of *An*. *arabiensis* does not. Tonnang *et al*. [12,35] projected *An*. *arabiensis* to reduce in range and population within Humid forest and Derived savanna in Nigeria, and concentrate more in Sahel, Sudan and Guinea savannas. Also, Drake and Beier [37] projected *An*. *arabiensis* to lose suitable habitats in Nigeria within Africa in 2050, instead of gaining. Although our projections agree with Tonnang *et al*. [12] regarding increased prevalence of *An*. *arabiensis* in Sahel, Sudan and Guinea savannas resulting from species invasion, they do not agree with reduction in suitable areas within Humid forest and Derived savanna, modelled to experience pervasive distribution of *An*. *arabiensis* in 2050s and more in 2070s [38,44]. Discrepancies of this sort, apart from the type of climate scenario used, may sometimes be attributed to differences in data sources, the general lack of the vector’s distributional data within area of interest, and the type of model used together with computational approaches [44,67,80,81]. Nevertheless, our model predictions of these *Anopheles* species’ fundamental niche characteristically conform to the realised geographical niche in Nigeria based on confirmed occurrence records [48]. MaxEnt model was able to use the included environmental variables to distinguish between presences and potentially unsampled locations [43,67] of the studied *Anopheles* species with average AUC of 0.7 (S1 File). This value of AUC score associated with the widespread of the species confirms the assertion of Lobo *et al*. [67] that, low AUC values denote the true generalist nature of the species distribution in a widespread species, which the probability of presence increases steadily with predictor values [38].

The north-eastward species shift in both RCP2.6 and RCP8.5 aligns with Peterson [34] who predicted west to east and west to south potential distributional shift of *An*. *gambiae* s.s. and *An*. *arabiensis* within Africa, respectively. Our model result also agrees with Tonnang *et al*. [35] who predicted that shifts in *An*. *gambiae* s.s. and *An*. *arabiensis* boundaries may occur southward and eastward of Africa rather than jumps into quite different climatic environments under climate change scenarios. The eastward shift, though tending more northward than southward is expected to be the case in Nigeria under both low and high emissions scenarios between 2041 and 2080. This does not overlook potential southward shift based on the north to south climate variation [50] and the location of the country within the tropical region. According to predictions by Moffett *et al*. [44], the abundance of *An*. *gambiae* and *An*. *arabiensis* is highest in West Africa (where Nigeria is located), as human population density and urbanisation are highly critical in malaria risk [34,44]. Rapid population expansion and increased deforestation through uncontrolled urbanisation and agricultural intensification [44] converge with energy consumption (dominated by fossil fuels) and poor or inadequate public health / healthcare infrastructure, alongside increased temperatures with high humidity, more recurrent temperature fluctuations from mean diurnal temperature range and longer high seasonal rainfall [33,34,40,82], to impel future increasing prevalence of the modelled malaria vectors in ecological subregions. Densely populated and urbanised Lagos state [83,84] within the humid forest of South West, and many other potentially populated / urbanised states with increased anthropogenic activities including Abia, Akwa Ibom, Anambra, Enugu, Imo, Ogun, Ondo, Osun, Oyo and Rivers states in the humid south, and Kano and Sokoto states within Sudan savanna in the North West are thus likely to be more vulnerable [20,76].

As the country will generally become warmer with high humidity and altered rainfall patterns, particularly under high emissions scenario, seasonal malaria transmission is expected to be higher and more unbalanced across the country [38,84,85], just as vector species prevalence increases and varies within ecolgical and regional zones (subregions) based on varying tropical climate conditions. This is because *Anopheles* mosquitoes thrive in regions with warm temperatures, humid conditions, and high rainfall [38,47,86,87] regulated by changes in background climate that alter the abundance, range (both latitude and altitude), distribution, and behaviour of malarial mosquitoes, and the life cycle of the malarial parasite, such that patterns of malaria changes [9,34,38,88,89]. The high magnitude of change in the species prevalence due to future changes in tropical climates within the tropical subregions, especially under high emissions scenario, lends credence to the proposition that, a little increase in temperature such as half degree centigrade increase can translate into a 30% to 100% increase in mosquito abundance [26,27]. This will most likely exacerbate malaria prevalence [90] by causing increases in the population at risk in areas where malaria presence is static in the future [91]—in line with World Health Organisation projections of more than 400 million people at risk of malaria by 2070 in Nigeria, under both high and low emissions scenarios [8,9,88].

## Conclusions

Anthropogenic climate change and land use dynamics are expected to increase the population and potential range of malaria vectors and exacerbate malaria burden. This study explored the effect of changing climate under low and high emissions scenarios on future composition and potential distribution of dominant malaria vector species in tropical subregions and administrative regions within the tropical country—Nigeria; using data of vectors known locations and environmental drivers of their occurrence and distribution, following algorithms defined in species distribution model—MaxEnt. Results show that *An*. *gambiae* s.l. and its two major siblings: *An*. *gambiae* s.s., and *An*. *arabiensis* will experience increased prevalence between 2041 and 2060, higher in 2061–2080 under high emissions scenario than low emission scenario in both 2041–2060 and 2061–2080; driven mainly by increasing and fluctuating temperature, longer seasonal tropical rainfall accompanied by drier phases, high humidity in dry season from precipitation during warm months, and inherent influence of rapid land use change. Humid forest and Derived savanna within all southern and most North Central states, with highest species invasion in Sahel and Sudan savannas, particularly in north-eastern states are likely to be most impacted. The predicted variability in species composition and distribution based on diversity in climate conditions in the tropical subregions across the country, may define the future spatial epidemiology of these vectors and possible malaria risk pattern. The impending high magnitude of change in prevalence of these dominant malaria vector species may lead to high malaria burden in 2050s, and higher in 2070s; especially under high emissions scenario, mostly dependent on past, current and future anthropogenic emissions and natural climate variability. Hence, there is need for more studies aimed at spatial estimation of future malaria prevalence at a finer scale with respect to vectors dynamics and distribution within the tropical subregions; and in relation to incremental differentials in climate and land use dynamics for the development of more robust adaptation strategies under global change.

## Supporting information

S1 Appendix Permission to publish content under CC BY 4.0 license. (PDF)

S1 Fig Environmental suitability for *An*. *gambiae* s.l. in Nigeria under baseline climate 1960–1990, low (RCP2.6) and high (RCP8.5) emissions scenarios 2041–2080. *Anopheles* species sampling points reprinted for illustrative purposes only from Okorie *et al*. [48] under a CC BY 4.0 license, with permission from PLOS ONE [38].
(TIF)

S2 Fig Environmental suitability for *An*. *gambiae* s.s. in Nigeria under baseline climate 1960–1990, low (RCP2.6) and high (RCP8.5) emissions scenarios 2041–2080. *Anopheles* species sampling points reprinted for illustrative purposes only from Okorie *et al*. [48] under a CC BY 4.0 license, with permission from PLOS ONE [38].
(TIF)

S3 Fig Environmental suitability for *An*. *arabiensis* in Nigeria under baseline climate 1960–1990 and, low (RCP2.6) high (RCP8.5) emissions scenarios 2041–2080. *Anopheles* species sampling points reprinted for illustrative purposes only from Okorie *et al*. [48] under a CC BY 4.0 license, with permission from PLOS ONE [38].
(TIF)

S1 File A file containing AUCs, tables for variable percent contributions, and jackknife test of variable importance to the MaxEnt model of *Anopheles gambiae* species. (XLSX)

## References

[1] World Health Organisation [Internet]. Malaria [Fact sheets]. 2019 March 27 [cited 2019 Apr 5]. Available at https://www.who.int/en/news-room/fact-sheets/detail/malaria.

[2] World Health Organisation [Internet]. The top 10 causes of death [Fact sheets]. 2018 May 24 [cited 2019 Apr 5]. Available at https://www.who.int/news-room/fact-sheets/detail/the-top-10-causes-of-death.

[3] World Health Organization. World Malaria Report 2018. Geneva: World Health Organization; 2018. 210p.

[4] Jain PC. Greenhouse effect and climate change: scientific basis and overview. *Renew Energ*. 1993; 3(4–5):403–420. 10.1016/0960-1481(93)90108-S.

[5] Gordy M. Urbanization and Land Use. In: Disaster Risk Reduction and the Global System SpringerBriefs in Climate Studies. Springer, Cham 2016 10.1007/978-3-319-41667-0_10.

[6] Ramasamy R, Surendran SN. Global climate change and its potential impact on disease transmission by salinity-tolerant mosquito vectors in coastal zones. *Front Physiol*. 2012; 3(198). 10.3389/fphys.2012.00198. PMC3377959 22723781

[7] Sutherst RW. Global Change and Human Vulnerability to Vector-Borne Diseases. *Clin Microbiol Rev*. 2004; 17(1):136–173. 10.1128/CMR.17.1.136-173.2004 14726459 PMC321469

[8] Climate and Health Country Profile– 2015, Nigeria. World Health Organization / United Nations Framework on Climate Change 2015.

[9] Martens WJ, Niessen LW, Rotmans J, Jetten TH, McMichael AJ. Potential Impact of Global Climate Change on Malaria Risk. *Environ Health Perspect*. 1995; 103(5):458–464. 10.1289/ehp.95103458 . 7656875 PMC1523278

[10] Fahey DW, Doherty SJ, Hibbard KA, Romanou A, Taylor PC. Physical drivers of climate change. In: Climate Science Special Report: Fourth National Climate Assessment, Volume I [Wuebbles DJ, Fahey DW, Hibbard KA, Dokken DJ, Stewart BC, Maycock TK (eds.)]. U.S. Global Change Research Program, Washington, DC, USA. 2017. pp. 73–113, 10.7930/J0513WCR

[11] Nelson GC, Bennett E, Berhe AA, Cassman K, DeFries R, Dietz T, et al Anthropogenic Drivers of Ecosystem Change: An Overview. *Ecol Soc*. 2006; 11(2):29 http://www.ecologyandsociety.org/vol11/iss2/art29/.

[12] Tonnang HEZ, Tchouassi DP, Juarez HS, Igweta LK, Djouaka RF. Zoom in at African country level: potential climate induced changes in areas of suitability for survival of malaria vectors. *Int J Health Geogr*. 2014; 13:12 10.1186/1476-072X-13-12 24885061 PMC4022448

[13] Riahi K, Rao S, Krey V, Cho C, Chirkov V, Fischer G, et al. RCP 8.5—A scenario of comparatively high greenhouse gas emissions. *Climatic Change*. 2011; 109:33–57. 10.1007/s10584-011-0149-y

[14] Meinshausen M, Smith SJ, Calvin K, Daniel JS, Kainuma ML, Lamarque JF, et al. The RCP greenhouse gas concentrations and their extensions from 1765 to 2300. *Clim Chang*. 2011; 109:213–241. 10.1007/s10584-011-0156-z

[15] van Vuuren DP, Edmonds J, Kainuma M, Riahi K, Thomson A, Hibbard K, et al The representative concentration pathways: an overview. *Clim Chang*. 2011; 109:5–31. 10.1007/s10584-011-0148-z.

[16] Van Vuuren DP, Stehfest E, den Elzen MGJ, Kram T, van Vliet J, Deetman S, et al RCP2.6: exploring the possibility to keep global mean temperature change below 2°C. *Clim Chang*. 2011; 109:95–116. 10.1007/s10584-011-0152-3.

[17] Fuss S, Canadell JG, Peters GP, Tavoni M, Andrew RM, Ciais P, et al Betting on negative emissions. *Nat Clim Chang*. 2014; 4(10):850–853.

[18] Hayhoe K, Edmonds J, Kopp RE, LeGrande AN, Sanderson BM, Wehner MF, et al. Climate models, scenarios, and projections. In: Climate Science Special Report: Fourth National Climate Assessment, Volume I [Wuebbles DJ, Fahey DW, Hibbard KA, Dokken DJ, Stewart BC, Maycock TK (eds.)]. U.S. Global Change Research Program, Washington, DC, USA. 2017. pp. 133–160, 10.7930/J0WH2N54

[19] Weber T, Haensler A, Rechid D, Pfeifer S, Eggert B, Jacob D. Analyzing regional climate change in Africa in a 1.5, 2, and 3°C global warming world. *Earth’s Future*. 2018; 6:643–655. 10.1002/2017EF000714.

[20] Federal Ministry of Environment of the Federal Republic of Nigeria. First national communication on climate change. Federal Ministry of Environment, Abuja: MEFRN; 2003.

[21] Khasnis AA, Nettleman MD. REVIEW ARTICLE: Global Warming and Infectious Disease. *Arch Med Res*. 2005; 36:689–696. 10.1016/j.arcmed.2005.03.041 . 16216650

[22] Ostfeld RS. Climate change and the distribution and intensity of infectious diseases. *Ecology*. 2009; 90(4):903–905. 19449683 10.1890/08-0659.1

[23] Riede JO, Posada R, Fink AH, Kaspar F. What’s on the 5th IPCC Report for West Africa? In: Yaro J., Hesselberg J. (eds) Adaptation to Climate Change and Variability in Rural West Africa. Springer, Cham 2016.

[24] Carabine E, Lemma A, Overseas Development Institute (ODI). The IPCC’s Fifth Assessment Report: What’s in it for Africa. Overseas Development Institute and Climate and Development Knowledge Network 2014.

[25] Federal Ministry of Environment of the Federal Republic of Nigeria. Second national communication on climate change. Federal Ministry of Environment, Abuja: MEFRN; 2014.

[26] Patz JA, Olson SH, Uejio CK, Gibbs HK. Disease emergence from global climate and land use change. *Med Clin N Am*. 2008; 92:1473–1491. 10.1016/j.mcna.2008.07.007 19061763

[27] Myers SS, Patz JA. Emerging Threats to Human Health from Global Environmental Change. *Annu Rev Environ Resour*. 2009; 34(1):223–252. 10.1146/annurev.environ.033108.102650

[28] Arndt C, Strzepeck K, Tarp F, Thurlow J, Fant IV C, Wright L. Adapting to climate change: an integrated biophysical and economic assessment for Mozambique. *Sustain Sci*. 2011; 6:7–20. 10.1007/s11625-010-0118-9 30174756 PMC6106621

[29] Rosenzweig C, Arnell NW, Ebi KL, Lotze-Campen H, Raes F, Rapley C, et al Assessing inter-sectoral climate change risks: the role of ISIMIP. *Environ*. *Res*. *Lett*. 2017; 12(010301). 10.1088/1748-9326/12/1/010301.

[30] Smith BA, Ruthman T, Sparling E, Auld H, Comer N, Young I, et al A risk modeling framework to evaluate the impacts of climate change and adaptation on food and water safety. *Food Res Int*. 2015; 68:78–85. 10.1016/j.foodres.2014.07.006.

[31] Legese W. Climate Change Indication and Projection Over Bale Highlands, Southeastern Ethiopia. *J Climatol Weather Forecasting*. 2017; 5: 212 10.4172/2332-2594.1000212

[32] Ren Z, Wang D, Ma A, Hwang J, Bennett A, Sturrock HJ, et al. Predicting malaria vector distribution under climate change scenarios in China: Challenges for malaria elimination. *Sci Rep*. 2016; 6(20604). 10.1038/srep20604 26868185 PMC4751525

[33] Onyabe DY, Conn JE. The Distribution of Two Major Malaria Vectors, *Anopheles gambiae* and *Anopheles arabiensis*, in Nigeria. *Mem Inst Oswaldo Cruz*, Rio de Janeiro. 2001; 96(8):1081–1084. 10.1016/s0035-9203(03)80045-7 . 11784926

[34] Peterson AT. Shifting suitability for malaria vectors across Africa with warming Climates. *BMC Infect Dis*. 2009; 9:59 10.1186/1471-2334-9-59 . 19426558 PMC2694813

[35] Tonnang HEZ, Richard YM, Kangalawe, Yanda PZ. Predicting and mapping malaria under climate change scenarios: the potential redistribution of malaria vectors in Africa. *Malar J*. 2010; 9:111 10.1186/1475-2875-9-111 20416059 PMC2873524

[36] Afrane YA, Githeko AK, Yan G. The Ecology of *Anopheles* Mosquitoes under Climate Change: Case Studies from the Effects of Environmental Changes in East Africa Highlands. *Ann N Y Acad Sci*. 2012; 1249:204–210. 10.1111/j.1749-6632.2011.06432.x 22320421 PMC3767301

[37] Drake JM, Beier JC. Ecological niche and potential distribution of *Anopheles arabiensis* in Africa in 2050. *Malar J*. 2014; 13:213 10.1186/1475-2875-13-213 24888886 PMC4066281

[38] Akpan GE, Adepoju KA, Oladosu OR, Adelabu SA. Dominant malaria vector species in Nigeria: Modelling potential distribution of *Anopheles gambiae* sensu lato and its siblings with MaxEnt. *PLoS ONE*. 2018; 13(10): e0204233 10.1371/journal.pone.0204233 30281634 PMC6169898

[39] CDC. Anopheles Mosquitoes [Internet]. Centers for Disease Control and Prevention: Global Health—Division of Parasitic Diseases and Malaria. c2015 [cited 2018 Jun 20]. Available from: http://www.cdc.gov/malaria/about/biology/mosquitoes/.

[40] Lindsay SW, Parson L, Thomas CJ. Mapping the ranges and relative abundance of the two principal African malaria vectors, *Anopheles gambiae* sensu stricto and *An*. *arabiensis*, using climate data. Proc R Soc London B. 1998; 265:847–854. 10.1098/rspb.1998.0369 PMC1689061 9633110

[41] Gillies MT, De Meillon B. The Anophelinae of Africa south of the Sahara (Ethiopian Zoogeographical Region). In Publications of the South African Institute for Medical Research Johannesburg. 1968 p. 54.

[42] Elith J, Phillips SJ, Hastie T, Dudík M, Chee YE, Yates CJ. A statistical explanation of MaxEnt for ecologists. *Diversity Distrib*. 2011; 17:43–57. 10.1111/j.1472-4642.2010.00725.x.

[43] Merow C, Smith MJ, Silander JA Jr. A practical guide to MaxEnt for modeling species’ distributions: what it does, and why inputs and settings matter. *Ecography*. 2013; 36(10):1058–1069. 10.1111/j.1600-0587.2013.07872.x.

[44] Moffett A, Shackelford N, Sarkar S. Malaria in Africa: Vector Species’ Niche Models and Relative Risk Maps. *PLoS ONE*. 2007; 2(9): e824 10.1371/journal.pone.0000824 . 17786196 PMC1950570

[45] Blackburn JK. Integrating Geographic Information Systems and Ecological Niche Modeling into Disease Ecology: A Case Study of *Bacillus anthracis* in the United States and Mexico In O'Connell KP, Skowronski EW, Bakanidze L, Sulakvelidze A (Eds.). Emerging and Endemic Pathogens. Vol 00. Dordrecht: NATO Science for Peace and Security Series A: Chemistry and Biology. Springer; 2010 p. 59–88. 10.1007/978-90-481-9637-1_7.

[46] Levine RS, Peterson AT, Benedict MQ. Geographic and ecologic distributions of the *Anopheles gambiae* complex predicted using a genetic algorithm. *Am J Trop Med Hyg*. 2004; 70: 105–109. . 14993618

[47] Kulkarni MA, Desrochers RE, Kerr JT. High Resolution Niche Models of Malaria Vectors in Northern Tanzania: A New Capacity to Predict Malaria Risk? *PLoS ONE*. 2010; 5(2):e9396 10.1371/journal.pone.0009396 . 20195366 PMC2827547

[48] Okorie PN, McKenzie FE, Ademowo OG, Bockarie M, Kelly-Hope L. Nigeria *Anopheles* Vector Database: An Overview of 100 Years’ Research. *PLoS ONE*. 2011; 6(12): e28347 10.1371/journal.pone.0028347 . 22162764 PMC3230596

[49] FAO Soils Bulletin 73. Agro-Ecological Zoning Guidelines. Soil Resources, Management and Conservation Service FAO Land and Water Development Division. Food and Agriculture Organization of the United Nations, Rome 1996 Retrieved from http://www.fao.org/docrep/w2962e/w2962e-03.htm#P229_15540.

[50] Climates to travel [Internet]. World climate guide: CLIMATE—NIGERIA. [Cited 2018 Jun 20]. Available from: https://www.climatestotravel.com/climate/nigeria.

[51] Metz HC (Ed.). Nigeria: A Country Study [Internet]. Washington: GPO for the Library of Congress. c1991 [cited 2018 Jun 20]. Available from: http://countrystudies.us/nigeria/33.htm.

[52] USAID. Nigeria Environmental Analysis Final Report. Biodiversity and Sustainable Forestry (BIOFOR) Indefinite Quantity Contract (IQC); 2002. USAID BIOFOR IQC No.: LAG-I-00-99-00013-00.

[53] United Nations, Department of Economic and Social Affairs, Population Division (UN-DESA). World Population Prospects: The 2017 Revision, Key Findings and Advance Tables. 2017; Working Paper No. ESA/P/WP/248.

[54] Owoseye A. World Malaria Day: Nigerians Warned to Stop Using Chloroquine for Malaria Treatment [Internet]. Premium Times. 2017 Apr 25 [cited 2018 Jun 20]. Available from: http://allafrica.com/stories/201704260043.html.

[55] Worldometers.info [Internet]. Dadax; Dover, Delaware, U.S.A.: Nigeria Population (LIVE); c2018 [cited 2018 Jun 20]. Available from: http://www.worldometers.info/world-population/nigeria-population/.

[56] National Malaria Control Programme, suNMaP, World Health Organization and the INFORM Project. A description of the epidemiology of malaria to guide the planning of control in Nigeria. A report prepared for the Federal Ministry of Health, Nigeria, the Roll Back Malaria Partnership and the Department for International Development, UK 2013 11.

[57] WorldClim—Global Climate Data. Free climate data for ecological modelling and GIS. [Cited 2017 Oct 6]. Available from: http://www.worldclim.org/version1.

[58] Ritchie H, Roser M. CO₂ and other Greenhouse Gas Emissions [Internet]. Our World in Data. c2018 [cited 2018 Jun 20]. Available from: https://ourworldindata.org/co2-and-other-greenhouse-gas-emissions.

[59] World Meteorological Organization (WMO). WMO Guidelines on the Calculation of Climate Normals. WMO 2017; 2017 edition.

[60] Tappan GG, Cushing WM, Cotillon SE, Mathis ML, Hutchinson JA, Dalsted KJ. West Africa Land Use Land Cover Time Series: U.S. Geological Survey data release [Internet]. c2016 [cited 2017 Oct 6]. Available from: 10.5066/F73N21JF.

[61] CGIAR-CSI. SRTM 90m Digital Elevation Data [Internet]. The CGIAR Consortium for Spatial Information (CGIAR-CSI). C2017 [cited 2017 Oct 6]. Available from: http://srtm.csi.cgiar.org/SELECTION/inputCoord2.asp.

[62] Phillips SJ, Anderson RP, Schapire RE. Maximum entropy modeling of species geographic distributions. *Ecol Model*. 2006; 190:231–259. 10.1016/j.ecolmodel.2005.03.026.

[63] Peterson AT. Ecologic Niche Modeling and Spatial Patterns of Disease Transmission. *Emerg Infect Dis*. 2006; 12(12):1822–1826. 10.3201/eid1212.060373 17326931 PMC3291346

[64] Phillips SJ. A Brief Tutorial on Maxent. 2017. Available from: http://biodiversityinformatics.amnh.org/open_source/maxent/.

[65] Young N, Carter L, Evangelista P. A MaxEnt Model v3.3.3e Tutorial (ArcGIS v10). Natural Resource Ecology, Colorado State University, USA 2011.

[66] Hanley JA, McNeil BJ. The meaning and use of the area under a Receiver Operating Characteristic (ROC) curve. *Radiology*, 1982; 143:29–36. 10.1148/radiology.143.1.7063747 . 7063747

[67] Lobo JM, Jiménez-Valverde A, Real R. AUC: a misleading measure of the performance of predictive distribution models. *Global Ecol Biogeogr*. 2008; 17(2):145–151. 10.1111/j.1466-8238.2007.00358.x.

[68] Fourcade Y, Engler JO, Rӧdder D, Secondi J. Mapping Species Distributions with MAXENT Using a Geographically Biased Sample of Presence Data: A Performance Assessment of Methods for Correcting Sampling Bias. *PLoS ONE*. 2014; 9(5): e97122 10.1371/journal.pone.0097122 . 24818607 PMC4018261

[69] Baldwin RA. Use of Maximum Entropy Modeling in Wildlife Research. *Entropy*. 2009; 11(4):854–866. 10.3390/e11040854.

[70] Chefaoui RM, Lobo JM. Assessing the effects of pseudo-absences on predictive distribution model performance. *Ecol Model*. 2008; 210:478–486.

[71] Altamiranda-Saavedra M, Arboleda S, Parra JL, Peterson AT, Correa MM. Potential distribution of mosquito vector species in a primary malaria endemic region of Colombia. *PLoS ONE*. 2017; 12(6): e0179093 Available from: 10.1371/journal.pone.0179093 28594942 PMC5464628

[72] Dimitrov NB, Morton DP. Combinatorial Design of a Stochastic Markov Decision Process In: Saltzman MJ, Chinneck JW, Kristjansson B, editors. Operations Research and Cyber-Infrastructure. New York: Springer; 2009 P. 167–193. 10.1007/978-0-387-88843-9.

[73] IPCC. The Regional Impacts of Climate Change: An Assessment of Vulnerability, (Eds Watson R. T., Zinyowera M. C., and Moss R. H.), Cambridge University Press, Cambridge, UK 1998.

[74] Zeng W, Cui X, Liu X, Cui H, Wang P. “Remote Sensing and GIS for Identifying and Monitoring the Environmental Factors Associated with Vector-borne Disease: An Overview.” 2006 IEEE International Symposium on Geoscience and Remote Sensing, Denver, CO, USA; 2006 pp. 1443–1446. 10.1109/IGARSS.2006.372

[75] Siteti MC, Injete SD, Wanyonyi WA. Malaria parasite species prevalence and transmission dynamics at selected sites in the Western highlands of Kenya. *CHRISMED J Health Res*. 2016; 3:45–50.

[76] Tolulope O. Spatio–Temporal Clustering of Malaria Morbidity in Nigeria (2004–2008). *J Sci Res*. 2014; 13:99–113.

[77] Ayala D, Costantini C, Ose K, Kamdem GC, Antonio-Nkondjio C, Agbor J, et al Habitat suitability and ecological niche profile of major malaria vectors in Cameroon. *Malar J*. 2009; 8:307 10.1186/1475-2875-8-307 20028559 PMC2805691

[78] Okwa OO, Akinmolayan FI, Carter V, Hurd H. Transmission dynamics of malaria in four selected ecological zones of Nigeria in the rainy season. *Ann Afr Med*, 2009; 8(1):1–9. 10.4103/1596-3519.55756 . 19762999

[79] Olayemi IK, Ande AT, Odeyemi MO, Ibemesi G Emmanuel R. Temporal Ecologic Adaptability of the Principal Vector of Malaria, *Anopheles gambiae* s.l. (Diptera: Culicidae), in Northcentral Nigeria. *App Sci Report*. 2014; 5(3):110–117. 10.15192/PSCP.ASR.2014.1.3.110117

[80] Pock Tsy JML, Duchemin JB, Marrama L, Rabarison P, Le Goff G, Rajaonarivelo V, Robert V. Distribution of the species of the *Anopheles gambiae* complex and first evidence of *Anopheles merus* as a malaria vector in Madagascar. *Malar J*. 2003; 2:33 10.1186/1475-2875-2-33 14609436 PMC269986

[81] Coetzee M. Distribution of the African malaria vectors of the *Anopheles*. *Am J Trop Med Hyg*. 2004; 70:103–104. . 14993617

[82] Phillips SJ, Dudík M. Modeling of species distributions with Maxent: new extensions and a comprehensive evaluation. *Ecography*. 2008; 31:161–175.64.

[83] Marais EA, Jacob DJ, Wecht K, Lerot C, Zhang L, Yu K, Kurosu TP, Chance K, Sauvage B. Anthropogenic emissions in Nigeria and implications for atmospheric ozone pollution: A view from space. *Atmos Environ*. 2014; 99: 32–40. 10.1016/j.atmosenv.2014.09.055.

[84] United Nations. World urbanization prospects: the 2003 revision. Data, tables and highlights. New York: United Nations 2004.

[85] Oyewole IO, Ibidapo CA, Oduola AO, Obansa JB, Awolola TS. Anthropophilic mosquitoes and malaria transmission in a tropical rain forest area of Nigeria. *acta SATECH*. 2005; 2(1):6–10.

[86] University Corporation for Atmospheric Research (UCAR) [Internet]. Climate Change and Vector-Borne Disease. c2011 [cited 2018 Jun 20]. Available from: https://scied.ucar.edu/longcontent/climate-change-and-vector-borne-disease.

[87] Adepoju KA, Akpan GE. Historical Assessment of Malaria Hazard and Mortality in Nigeria—Cases and Deaths: 1955–2015. *Int J Environ Bioener*. 2017; 12(1):30–46.

[88] IPCC. Climate Change 1995 Impacts, Adaptations and Mitigation of Climate Change: Scientific-Technical Analyses Contribution of Working Group II to the Second Assessment Report of the Intergovernmental Panel on Climate Change. (Watson RT, Zinyowera MC, Moss RH, Dokken DJ, Eds.) New York, USA: Press Syndicate of the University of Cambridge 1995.

[89] Rogers DJ, Randolph SE. The global spread of malaria in a future, warmer world. *Science*. 2000; 289:1763–1765. . 10976072 10.1126/science.289.5485.1763

[90] Pascual M, Ahumada JA, Chaves LF, Rodo X, Bouma M. Malaria resurgence in the East African highlands: temperature trends revisited. Proceedings of the National Academy of Sciences of the United States of America. 2006; 103(15):5829–5834 10.1073/pnas.0508929103 . 16571662 PMC1416896

[91] Dutta S. Malaria Epidemiology on Jalpaiguri District Applying Remote Sensing and Geographic Information System. Centre for Remote Sensing Application, North Bengal University 2006.

