# Supplementary figures and images for: Potential distribution of dominant malaria vector species in tropical region under climate change scenarios
Source: PLoS One. 2019 Jun 19;14(6):e0218523. doi: 10.1371/journal.pone.0218523 (PMC6583992; doi:10.1371/journal.pone.0218523)

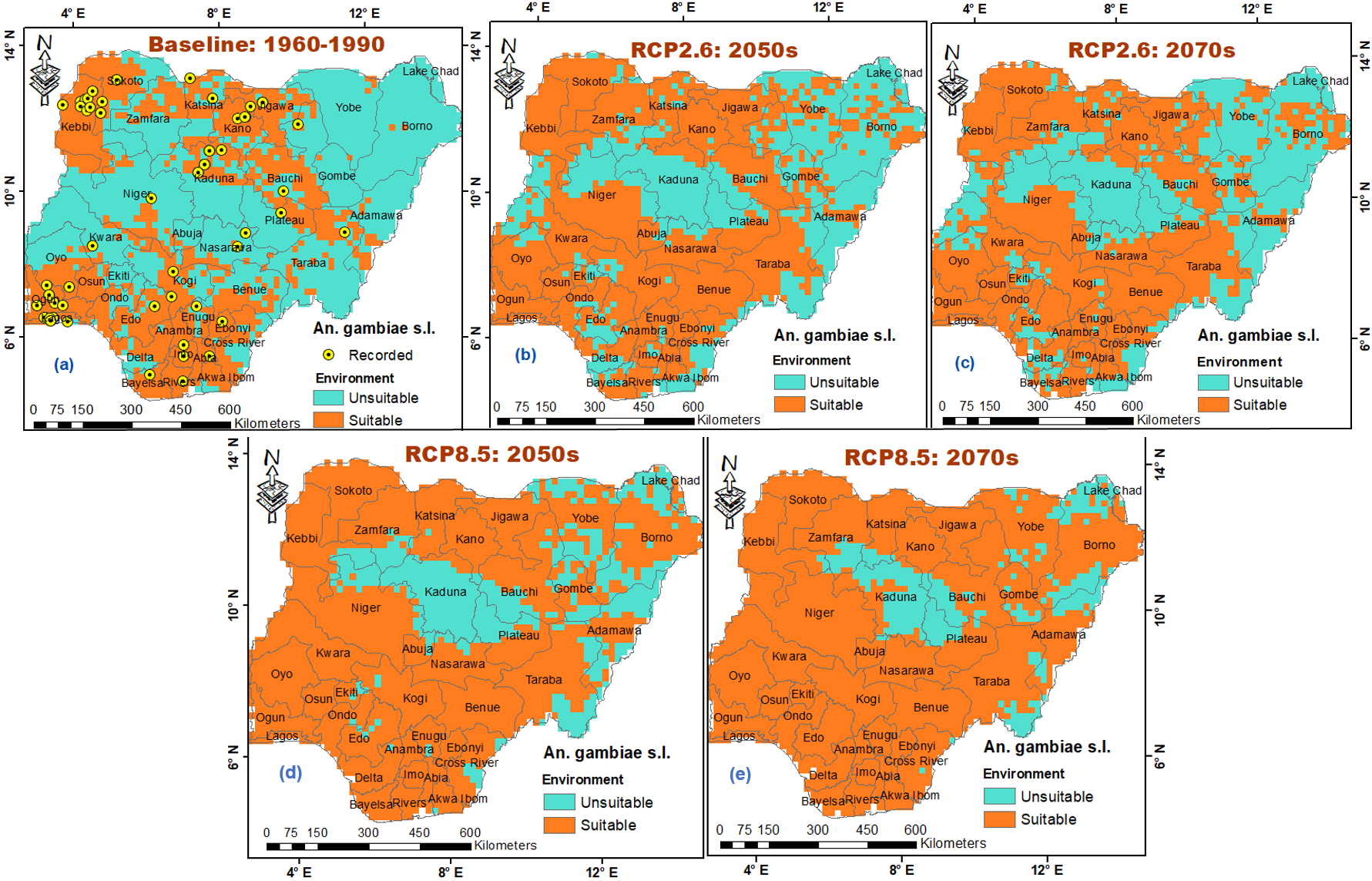

Supplement: S1 Fig — Anopheles species sampling points reprinted for illustrative purposes only from Okorie et al. [48] under a CC BY 4.0 license, with permission from PLOS ONE [38]. (TIF) [file pone.0218523.s002.tif]

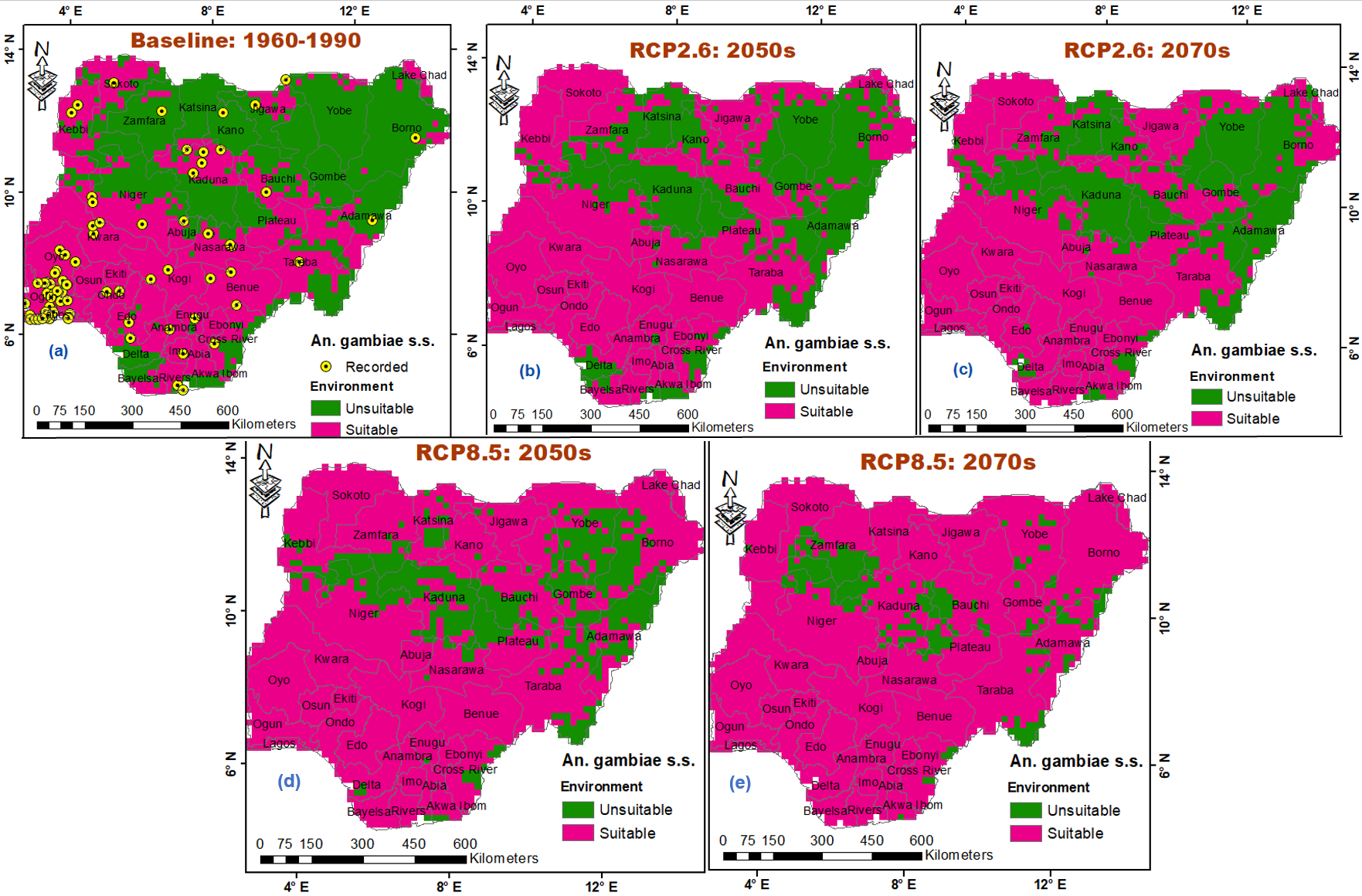

Supplement: S2 Fig — Anopheles species sampling points reprinted for illustrative purposes only from Okorie et al. [48] under a CC BY 4.0 license, with permission from PLOS ONE [38]. (TIF) [file pone.0218523.s003.tif]

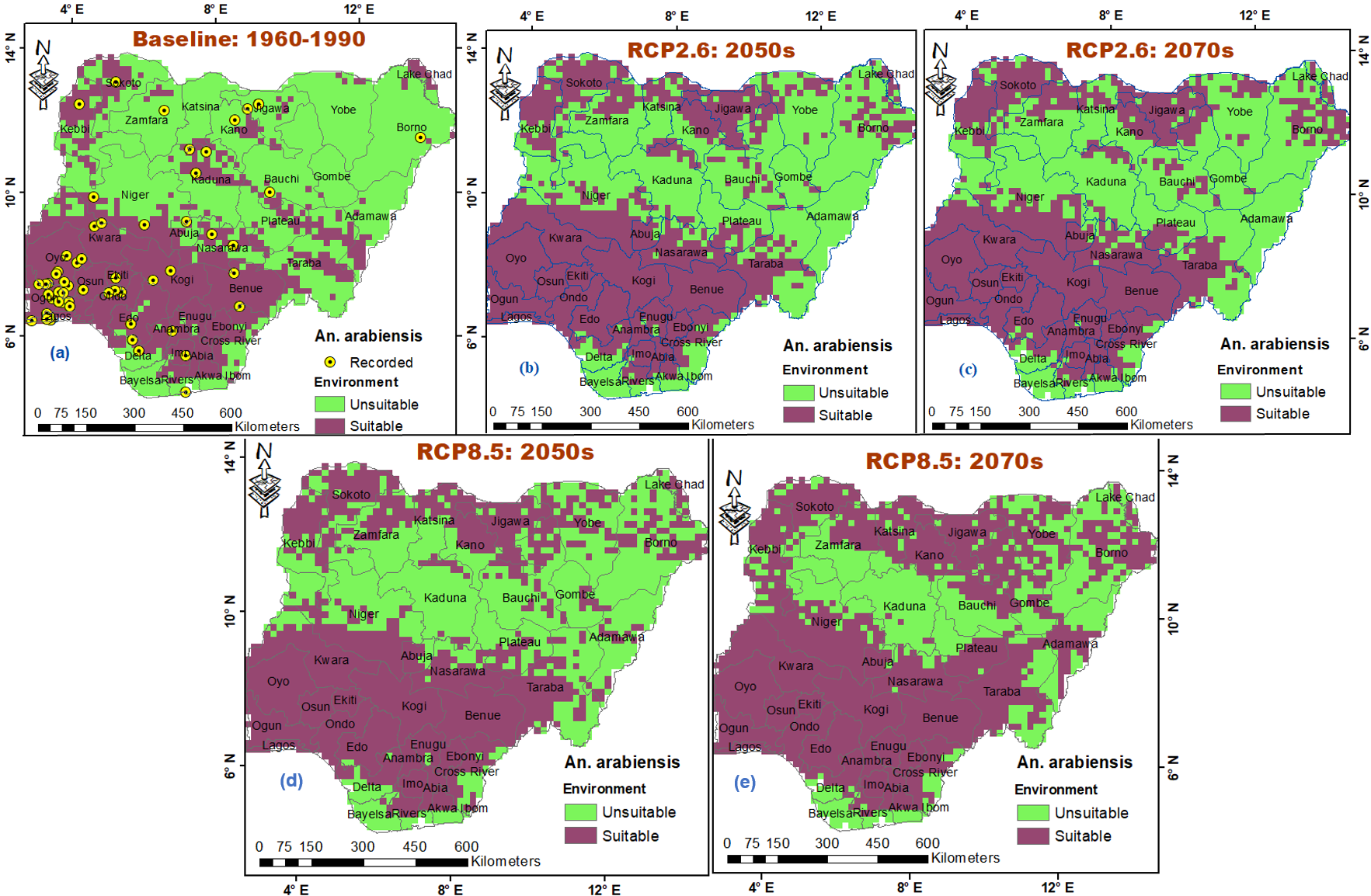

Supplement: S3 Fig — Anopheles species sampling points reprinted for illustrative purposes only from Okorie et al. [48] under a CC BY 4.0 license, with permission from PLOS ONE [38]. (TIF) [file pone.0218523.s004.tif]
