# Supplementary material for: Potential distribution of dominant malaria vector species in tropical region under climate change scenarios
Source: PLoS One. 2019 Jun 19;14(6):e0218523. doi: 10.1371/journal.pone.0218523 (PMC6583992; doi:10.1371/journal.pone.0218523)
Supplement: S1 Appendix — (PDF) [file pone.0218523.s001.pdf]

| | |
|---|---|
| Subject | **FW: Request for Permission to Publish Content under CC-BY License** |
| From | Maija Mallula <plosone@plos.org> |
| Sender | <noreply@salesforce.com> |
| To | godwin.akpan@arcsstee.org.ng <godwin.akpan@arcsstee.org.ng> |
| Date | 23.05.2018 19:26 |

Dear Dr. Akpan,

Thank you for reaching out to us regarding the re-use of PLOS ONE content. All PLOS ONE content is published under the CC BY 4.0 license and is freely available online. Anyone is permitted to access, download, copy, distribute, and re-use these materials in any way, even commercially, with proper attribution. As such, you are not required to obtain permission to use the georeferenced locations of Nigeria Anopheles species and you are welcome to re-use them with proper attribution.

Please let me know if you have any additional questions or concerns.

Kind regards,
Maija Mallula

PLOS | OPEN FOR DISCOVERY
Maija Mallula | Publications Assistant, PLOS ONE
1160 Battery Street, Suite 225, San Francisco, CA 94111
plosone@plos.org | | Main +1 415-624-1200 | Fax +1 415-546-4090

Case Number: 05776797

-------------- Original Message --------------
From: PLOS [plos@plos.org]
Sent: 5/22/2018
To: plosone@plos.org
Subject: FW: Request for Permission to Publish Content under CC-BY License

From: Godwin Akpan <profjedidiah@yahoo.com>
Sent: Tuesday, May 22, 2018 6:18 AM
To: PLOS <PLOS@plos.org>
Subject: Request for Permission to Publish Content under CC-BY License

Dear Sir/Madam,

I hope this email finds you well. I am writing to request permission for the open-access journal PLOS ONE to publish georeferenced locations of Nigeria Anopheles species for illustrations on my modelled suitability / distribution maps of some Anopheles species under the Creative Commons Attribution License (CCAL) CC BY 4.0 as attached below.

Thank you for your time and consideration.

Best regards,

Godwin Akpan
Graduate Research Scientist
African Regional Centre for Space Science &
Technology Education in English (ARCSSTEE)
Obafemi Awolowo University, Ile-Ife (OAU) Affiliated to United Nations;
and Centre for Space Research and Applications (CESRA),
The Federal University of Technology, Akure (FUTA).

Phone: +2348065289311
Email: godwin.akpan@arcsstee.org.ng<mailto:godwin.akpan@arcsstee.org.ng>

PLOS | OPEN FOR DISCOVERY
Maija Mallula | Publications Assistant, PLOS ONE
1160 Battery Street, Suite 225, San Francisco, CA 94111
plosone@plos.org | Main +1 415-624-1200 | Fax +1 415-546-4090
ref:_00DU0Ifis._5000BjtPH1:ref
